# Synthesis of Synthetic Musks: A Theoretical Study Based on the Relationships between Structure and Properties at Molecular Scale

**DOI:** 10.3390/ijms24032768

**Published:** 2023-02-01

**Authors:** Xixi Li, Hao Yang, Yuanyuan Zhao, Qikun Pu, Tingzhi Xu, Rui Li, Yu Li

**Affiliations:** 1 State Key Laboratory of Environmental Criteria and Risk Assessment, Chinese Research Academy of Environmental Sciences, Beijing 100012, China; 2 State Environmental Protection Key Laboratory of Ecological Effect and Risk Assessment of Chemicals, Chinese Research Academy of Environmental Sciences, Beijing 100012, China; 3 Northern Region Persistent Organic Pollution Control (NRPOP) Laboratory, Faculty of Engineering and Applied Science, Memorial University, St. John’s, NL A1B 3X5, Canada; 4 MOE Key Laboratory of Resources and Environmental Systems Optimization, North China Electric Power University, Beijing 102206, China

**Keywords:** synthesizability, machine learning, QSAR, DFT, human health risk

## Abstract

Synthetic musks (SMs), as an indispensable odor additive, are widely used in various personal care products. However, due to their physico-chemical properties, SMs were detected in various environmental media, even in samples from arctic regions, leading to severe threats to human health (e.g., abortion risk). Environmentally friendly and functionally improved SMs have been theoretically designed in previous studies. However, the synthesizability of these derivatives has barely been proven. Thus, this study developed a method to verify the synthesizability of previously designed SM derivatives using machine learning, 2D-QSAR, 3D-QSAR, and high-throughput density functional theory in order to screen for synthesizable, high-performance (odor sensitivity), and environmentally friendly SM derivatives. In this study, three SM derivatives (i.e., D52, D37, and D25) were screened and recommended due to their good performances (i.e., high synthesizability and odor sensitivity; low abortion risk; and bioaccumulation ability in skin keratin). In addition, the synthesizability mechanism of SM derivatives was also analyzed. Results revealed that high intramolecular hydrogen bond strength, electrostatic interaction, qH^+^ value, energy gap, and low E_HOMO_ would lead to a higher synthesizability of SMs and their derivatives. This study broke the synthesizability bottleneck of theoretically designed environment-friendly SM derivatives and advanced the mechanism of screening functional derivatives.

## 1. Introduction

Synthetic musks (SMs), as a kind of additive with a particular smell, have been widely used in perfume, shower gel, shampoo, lotion, shampoo, shaving cream, soap, deodorant, sunscreen, nail polish, hair oil, hair dye, lip balm, aftershave softener, air freshener, washing powder, and other personal care products in daily life to enhance the fragrance and mask odors [1]. SMs contain various compounds and have been categorized into four categories (i.e., nitromusks, polycyclic, macrocyclic, and cycloaliphatic). Polycyclic musks such as galaxolide (HHCB) and tonalide (AHTN) have been classified as high-yield chemicals [2]. Specifically, the annual output of HHCB in the United States reached about 4536 tons in 2015 [3]. After using personal care products, they entered the environment through water and air, thus polluting the environment. SMs can also cause secondary pollution problems because they cannot be completely degraded in their environments [2,3,4,5,6,7]. SMs have been reported in various environmental matrices, such as surface water [8,9], air [10], and soil [2,11], and HHCB was even detected in sediment samples from the Arctic [12]. Regarding their environmental properties, SMs are considered semi-volatile, lipophilic, bio-accumulative, and partially biodegradable [4,13,14]. The bioaccumulation abilities of HHCB and AHTN have called for extensive attention in recent years. HHCB and AHTN have been found to cause endocrine disruption and antiestrogenic activity in animals and humans [4,15] and have also been shown to accumulate in living tissues and blood with potential adverse health effects [16,17]. Furthermore, as an emerging pollutant, SMs have been proven to have endocrine-disrupting effects and developmental toxicity to embryonic development, which can potentially cause abortion risks in pregnant women [1,15,18]. Therefore, it is important to investigate and reduce the risk of SMs to the environment and human health.

The environmentally friendly molecular design has been widely used for the source control of emerging pollutants. Zhou et al. [19] designed a novel bee-friendly peptidomimetic insecticide based on 3D-QSAR. Zhao et al. [20] developed and screened four neonicotinoid insecticides with bidirectional selectivity. Specifically, these four insecticides had enhanced toxic effects on pests and grubs (increased by 1.44–12.58%), while the chronic sublethal impact on beneficial insects (i.e., bees and earthworms) slowed down (0.29–27.18%). Ren et al. [21] designed two LEV substitute molecules using levofloxacin (LEV) as the parent molecule. They found that the binding effects of LEV substitutes on B-G mutant proteins were increased by 13.24% and 19.40%, respectively. These results indicated that antibacterial drug resistance had improved, thus inhibiting its vertical gene transfer ability in humans. Fu et al. [22] designed and screened a fluoroquinolone substitute molecule with high biodegradability (+120.51%), improved functional properties (genotoxicity) (+13.66%), decreased bioaccumulation (−44.81%), and relieved human liver toxicity (−106.21%). In addition, a variety of SM derivatives, including 19 SM derivatives with improved functionality (strong odor sensitivity) [23], ten function-improved SM derivatives with reduced risk through dermal exposure [24], and 48 SM derivatives with lower abortion risk [18], were evaluated in our previous studies. The design of those environmentally friendly alternatives for emerging pollutants is generally based on theoretical methods. Although it has been confirmed from multiple perspectives, such as molecular functional characteristics, environmental characteristics, environmental existence, and environmental friendliness improvement mechanisms of emerging pollutant substitutes, none of these derivatives have been synthesized. It is undoubtedly a shortcoming for theoretically designed emerging pollutant derivatives. For this reason, compared with the commercially available emerging pollutants, the research on the synthesizability and mechanism of theoretically designed emerging pollutant substitute molecules is expected to make up for the above shortcomings to a certain extent. It can also provide technical support for effectively reducing development costs when put into experimental synthesis.

The synthesizable studies of environmentally friendly emerging contaminant derivatives were barely reported. Density functional theory (DFT) has been widely used to predict the positive frequency value of emerging pollutant derivatives to verify whether the designed molecules can exist in the environment [21,25,26,27]. However, the synthesizability of derivatives calculated by the DFT method has not been compared with their precursors; thus, the prediction is incomplete. Frey et al. [28] used a machine learning (ML) model to predict the synthesizability of two-dimensional metal carbides and nitrides and their precursors for the first time and screened 18 molecules and 111 phases from 56 MXenes and 792 MAX phases, respectively, with improved synthesis efficiency. By this means, this paper attempted to improve the prediction ability of accurately screening environmentally friendly SMs and their derivatives with improved functionality using DFT and ML methods.

In order to accurately verify the predicted synthesizability of SM derivatives, it is important to analyze the mechanism of the relationship between the molecular structures and descriptors and the synthesizability of SM derivatives. The integration of DFT and ML methods for predicting the synthesizability of SM derivatives is based on the comparative analysis of the topological parameters, electronic parameters, geometric parameters, and physico-chemical parameters of the SMs and SM derivatives. The sensitivity coefficient analysis was conducted using the two-dimensional quantitative structure-activity relationships (2D-QSAR) model for screening key feature molecular descriptors [29,30]. In addition, to further understand the influence of molecular structure on the synthesis of SM derivatives and to further guide the design of environmentally friendly artificial musk substitutes, the contour maps of a three-dimensional quantitative structure-activity relationship (3D-QSAR) model were introduced [31]. Coupling molecular force field information with key descriptors affecting the molecular synthesis of SM derivatives for analyzing the synthesis of SM derivatives has never been reported before. Intramolecular hydrogen bonds can directly affect the molecular stability of compounds [32]. The calculation of intramolecular hydrogen bonds can help to obtain high stability and synthesizability of new compounds due to their directional non-covalent interaction [33]. Thus, the theoretical analysis of intramolecular hydrogen bond theory can be used to analyze the synthesizability of SM derivatives. Therefore, this study investigated the mechanism analysis for the synthesizability of environmentally friendly SMs, which has never been tackled before.

The objective of this study is thus to summarize the environmentally friendly SM derivatives with improved functionalities described in the existing research, aiming to provide the primary mechanism analysis and synthetic strategy for the synthesizability of theoretically designed SM derivatives. This is the first attempt to combine DFT with multiple ML methods to accurately predict the synthesizability of SM derivatives. In-depth synthesizable mechanisms for designing environmentally friendly SMs were investigated by integrating the 2D-QSAR model, the 3D-QSAR model, and intramolecular hydrogen bond theory. This study constructed a cost-effective screening system for synthesizing theoretically designed SM derivatives, which is expected to provide theoretical guidance for replacing emerging pollutants and developing new materials.

## 2. Results and Discussion

### 2.1. Dimensionality Reduction of Descriptors of SM and SM Derivatives Using Pearson Correlation Coefficient Method

**Figure 1:**
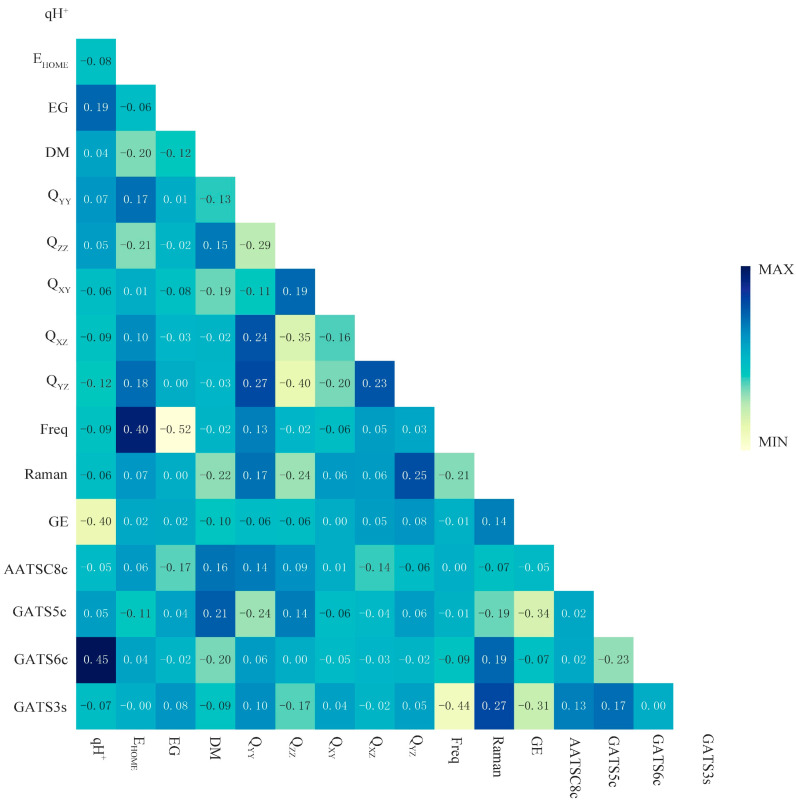
The Pearson correlation coefficient of SMs and SM derivatives after dimensionality reduction.

In this study, 1471 descriptors, including electronic parameters, structural parameters, topological parameters, physico-chemical parameters, and spectra of SMs and SM derivatives, were calculated by Gaussian 09 (Gaussian, Inc., Wallingford, CT, USA), PaDEL-Descriptor, and ChemBioDraw 12.0 software (PerkinElmer, Waltham, MA, USA). After eliminating the descriptors without values, there were 1022 descriptors left, which are provided in Supplementary Material II. These descriptors were loaded into the “python software (python 3.11.1, Holland) code package” for the Pearson correlation coefficient analysis. The correlation between descriptors was calculated. The characteristic descriptors of the PU machine learning model were screened by adjusting the classification threshold (which was set at *p* = 0.6 in this study), and the characteristic values with strong correlations were automatically eliminated. As shown in Figure 1, the number of molecular descriptors of SMs and SM derivatives was reduced from 1471 to 16 by the Pearson correlation coefficient method. The Pearson correlation coefficient (PCC) can be used to describe the strength of the linear relationship between each pair of descriptors. The relationship strength between two descriptors is considered low when the absolute value of PCC is <0.5 [34]. It can be seen from Figure 1 that the 16 key descriptors were screened out, including qH^+^, E_HOMO_, energy gap (EG), dipole moment (DM), Q_YY_, Q_ZZ_, Q_XY_, Q_XZ_, Q_YZ_, positive frequency (Freq), Raman, GE, AATSC8c, GATS5c, GATS6c, and GATS3s. The PCC of the key descriptors were all within a reasonable range, and the dataset also meets the training requirements of the bagging-PU model in this paper [28].

### 2.2. The Synthesizability Calculation of SM Derivatives

#### 2.2.1. Synthesizability Calculation of SM Derivatives Based on the Bagging-RF Algorithm

In this paper, the 88 SMs and SM derivatives retrieved were divided into positive and unlabeled samples, of which 11 commercial SMs were marked as positive samples. The remaining 77 theoretically designed and yet-to-be synthesized environmentally friendly SM derivatives were labelled as “unlabeled samples”. In each iteration of the PU machine learning process, some unlabeled samples can be randomly marked as unlabeled, non-synthesizable samples. In addition, the key descriptors screened by Pearson correlation coefficients were used to construct a random forest base classifier for predicting the synthesizability of unlabeled samples. The above PU machine learning procedure was repeated for 23 iterations, and the unlabeled, non-synthesizable samples were relabeled in each iteration. Furthermore, the synthesizability of an unlabeled sample was defined as the average of the RF classifier prediction scores for all samples except that sample. The SM derivatives with synthesizability greater than 0.5 were marked as an unlabeled synthesizable sample, and those with less than 0.5 were marked as an unlabeled non-synthesizable sample [28]. The model outputted the synthesizability of unlabeled synthesizable samples as an indicator representing their synthesizability. The 16 key descriptors screened were used to construct a PU ML prediction model for the synthesizability of SM derivatives based on the bagging-RF algorithm. The evaluation score of the built model was 0.705 (>0.7), indicating that the model has good predictive performance [28]. According to Table 1, the predicted synthesizability of 16 (i.e., D3, D5, D7, D8, D22, D25, D28, D33, D35, D37, D38, D50, D51, D52, D60, and D34) out of 77 unlabeled samples was greater than 0.5. So, the above 16 theoretically designed environment-friendly SM derivatives had strong synthesizability. This result provides important guidance for developing green SM derivatives in the future and simultaneously reduces the number of unlabeled samples by 79.22%, which minimizes the research cost of subsequent musk substitute molecules.

#### 2.2.2. Synthesizability Calculation of SM Derivatives Based on Bagging-ERT Algorithm

In this paper, bagging-ERT ML was used to predict the synthesis of SM derivatives. The Extremely Randomized Tree Classifier (ERT) was used in the replacement algorithm of the PU machine learning prediction model to prove that the synthesis of SM derivatives does not depend on specific predictive models. Eleven commercial SMs were selected as positive samples, and the remaining 77 theoretically designed environmentally friendly SM derivatives that have not yet been synthesized were used as unlabeled samples. The bagging-ERT algorithm for predicting the synthesizability of SM derivatives was constructed. The evaluation score of the built model was 0.727 (>0.7), indicating that the model has good predictive performance [28]. Among the 77 unlabeled samples, the predicted synthesizability of 16 unlabeled samples (i.e., D5, D8, D9, D11, D23, D25, D26, D29, D34, D35, D38, D51, D52, D53, D57, and D60) was greater than 0.5. Thus, the above 16 theoretically designed environment-friendly SM derivatives had strong synthesizable properties in the follow-up experimental development process (Table 2). Compared with the RF algorithm, 11 SMs substitute molecules (i.e., D7, D8, D22, D25, D28, D33, D34, D37, D50, D51, and D52) with a high synthesizable probability were screened out by both the bagging-RF and bagging-ERT algorithms. The synthesizability of these SM derivatives was ranked in the top 10 of the two prediction models. The deviations of synthesizability for D37, D8, D22, D34, D51, D28, and D52 in the bagging-RF and bagging-ERT prediction models were less than 10.13%, indicating that the constructed bagging-RF and bagging-ERT machine learning models both had high prediction accuracy.

#### 2.2.3. Synthesizability Calculation of SM Derivatives Based on Bagging-GBC Algorithm

**Figure 2:**
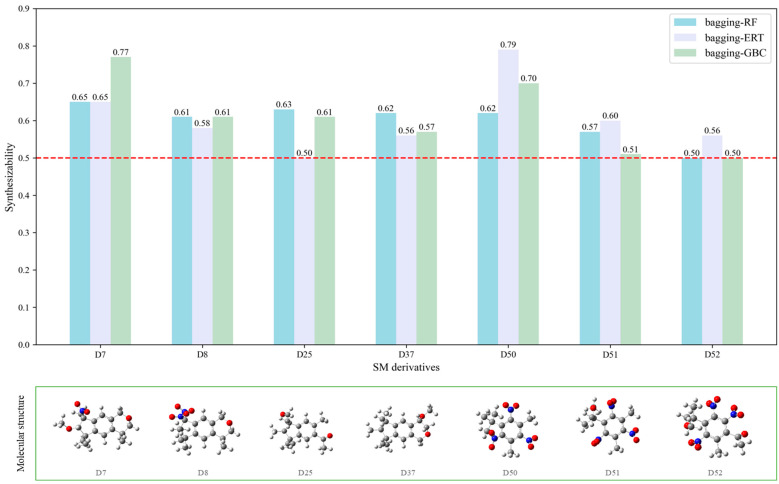
The synthesizability and molecular structures of synthesizable SM derivatives predicted by three PU models.

The gradient-boosting classifier (GBC) was selected as the basic classifier to construct the bagging-gradient-boosting classifier (bagging-GBC) model for further analyzing the molecular synthesis of SM derivatives. We aim to screen the SM derivatives with high predicted synthesizability that meet different models so that these screened environmentally friendly SM derivates could have the highest probability of synthesis. The synthesizability of the SM derivatives was predicted using the bagging-GBC algorithm. The constructed model showed an evaluation score of 0.770 (>0.7), indicating that the model has good predictive performance [28]. Table 3 shows the prediction results of the PU machine learning model for the molecular synthesizability of SM derivatives based on the bagging-GBC algorithm. The synthesizability of D6, D7, D8, D25, D32, D35, D37, D50, D51, and D52 is greater than 0.5. Seven SM derivatives (i.e., D7, D8, D25, D37, D50, D51, and D52) were screened and simultaneously met the requirements of synthesizable probability (greater than 0.5) predicted by bagging-GBC, bagging-ERT, and bagging-RF algorithms. The synthesizability and molecular structures of these seven SM derivatives are provided in Figure 2. The synthesizability of D7 and D50 predicted by three ML models was high. Specifically, the synthesizability of D7 predicted by the three models was all greater than 0.65, and the synthesizability of D50 predicted by the three models was greater than 0.62 (Table 3). In addition, the synthesizability of the seven SM derivatives ranked in the top 10 of the predicted values predicted by the bagging-GBC, bagging-ERT, and bagging-RF models, indicating the prediction accuracy of the three synthesizable prediction models has consistency (Table 4).

### 2.3. Evaluation of Environmental Risk and Functional Properties of SM Derivatives

The environmental risks and functional properties of seven SM derivatives screened by the bagging-GBC, bagging-ERT, and bagging-RF models were predicted by the 3D-QSAR models constructed by Li et al. [18,23,24] and EPI Suite 4.1 (U.S. Environmental Protection Agency, USA) software. The results are shown in Table 5. The abortion risk induced by SM derivatives was represented by the docking scores of SM derivatives to estrogen (1A52) and progesterone (1A28), respectively [24]. The docking score of SM derivatives to skin keratin proteins (4ZRY) was used to characterize the bioaccumulation of SM derivatives in humans [18]. The biotoxicity of SM derivatives was reflected by LC_50_ in the fish [23]. The functional property (i.e., odor sensitivity) of SM derivatives was represented by the binding energy of SM derivatives and human olfactory protein (OR5AN1).

It was found that D37, as a substitute molecule for HHCB, had a 7.18% improvement in its functional properties (odor sensitivity) compared with HHCB. The docking scores of D37 to 1A52 and 1A28 decreased, indicating that the abortion risk induced by D37 was alleviated. In addition, D37 had an improved LC_50_ value in fish and reduced bioaccumulation ability in skin keratin, indicating that the environmental risks of D37 were lower than those of its precursor, HHCB. Therefore, D37, with its high synthesizability and lower environmental impacts, can be an alternative for HHCB. According to Table 5, the odor sensitivity of D7 was increased, and its bioaccumulation ability and abortion risk were reduced. Moreover, the biological toxicity of D7 is one level lower than that of its precursor, HHCB [23]. Thus, D7 can be recommended as another substitute molecule for HHCB. Compared with the other six screened SM derivatives, the functional properties (odor sensitivity) of D52 were significantly improved (10.77%). For the design of SM alternatives with improved functional properties, D52 is the first choice as the substitute for its precursor, MK. Furthermore, D52 has almost equal bioaccumulation ability to MK but a lower abortion risk and lower biotoxicity. For PHAN, the odor sensitivity of D25 was increased by 7.65%, and the biotoxicity was significantly reduced (−743.48%). The predicted LibDock scores of D25 for two hormone proteins (i.e., progestogen and estrogen) were consistent with those of its parent molecule, PHAN, maintaining the same level of abortion risk. Nakata et al. [35] found that skin contact was the most important way for SMs to be absorbed into the human body. Thus, the bioaccumulation ability of SMs can be effectively reduced by inhibiting their entry into skin keratin. The bioaccumulation ability of D25 was decreased by 17.25%, indicating that D25 can be recommended as one of the environmentally friendly substitutes for PHAN, which is consistent with the conclusion provided by Li et al. [24].

### 2.4. Synthesizable Mechanism Analysis of SM Derivatives

#### 2.4.1. Sensitivity Analysis of the Synthesizability of SM Derivatives

To further analyze the influence of the 16 key descriptors (i.e., qH^+^, E_HOMO_, energy gap (EG), dipole moment (DM), Q_YY_, Q_ZZ_, Q_XY_, Q_XZ_, Q_YZ_, positive frequency (Freq), Raman, GE, AATSC8c, GATS5c, GATS6c, and GATS3s) screened by the Pearson correlation coefficient method in Section 3.1, the python software was used to output the correlation ranking between the 16 key descriptors and synthesizability. Then the sensitivity analysis of the top 50% most important key descriptors was analyzed by SPSS software to explore the important factors affecting the synthesis of SM derivatives. With the help of the “sklearn.feature_selection” package in the ML tool library scikit-learn, the relationship between the synthesizability of SM derivatives and 16 key descriptors in the bagging-RF, bagging-ERT, and bagging-GBC algorithms [36] was analyzed. The SelectKBest function was used to output the correlation ranking of 16 key descriptors in the three algorithm models (Table 6).

Raman, energy gap, qH^+^, dipole moment, positive frequency, Q_YY_, and E_HOMO_ are the top 50% of the key descriptors in three models; Q_XY_ is the top 50% of the key descriptors in two models; and AATSC8c ranks the top 50% of the descriptors of the key feature correlation of the bagging-GBC model. Therefore, this paper selected nine key eigenvalues (i.e., Raman, energy gap, qH^+^, dipole moment, positive frequency, Q_YY_, E_HOMO_, Q_XY_, and AATSC8c) for the sensitivity analysis. The above nine key descriptor values were used as independent variables, and the synthesizability of SM derivatives was used as a dependent variable for constructing three linear regression models by SPSS software. The correlation coefficients R of the bagging-RF, bagging-ERT, and bagging-GBC algorithms were 0.793, 0.825, and 0.805, respectively, all of which met the statistical requirements, and the Sig. of these models were all 0.000, passing the significance test [37]. The linear relationship between the synthesizability of SM derivatives and key descriptors was shown in Formula (1) (bagging-RF), Formula (2) (bagging-ERT), and Formula (3) (bagging-GBC). The coefficients of Raman, positive frequency, and E_HOMO_ are positive, indicating a positive correlation between these three descriptors and the synthesizability of SM derivatives. The coefficients of the energy gap, qH^+^, dipole moment, Q_YY_, Q_XY_, and AATSC8c are negative, indicating a negative correlation between these three descriptors and the synthesizability of SM derivatives.

(1) $$\begin{array}{cc} Y=0.64848 & +0.00114\times Raman-0.61591\times EG-0.57966\times qH^{+}\\ & -0.02471\times DM+0.00008\times Freq-0.00701\times Q_{YY}\\ & +0.54305\times E_{HOMO}-0.00033\times Q_{XY}-7.81030\times AATSC8c \end{array}$$

(2) $$\begin{array}{cc} Y=0.76458 & +0.00133\times Raman-0.82302\times EG-0.98987\times qH^{+}\\ & -0.04021\times DM+0.00014\times Freq-0.01438\times Q_{YY}\\ & +0.49688\times E_{HOMO}-0.00017\times Q_{XY}-17.60495\times AATSC8c \end{array}$$

(3) $$\begin{array}{cc} Y=0.75092 & +0.00170\times Raman-0.87826\times EG-1.19493\times qH^{+}\\ & -0.04326\times DM+0.00015\times Freq-0.01299\times Q_{YY}\\ & +0.82854\times E_{HOMO}-0.00036\times Q_{XY}-10.28994\times AATSC8c \end{array}$$

**Figure 3:**
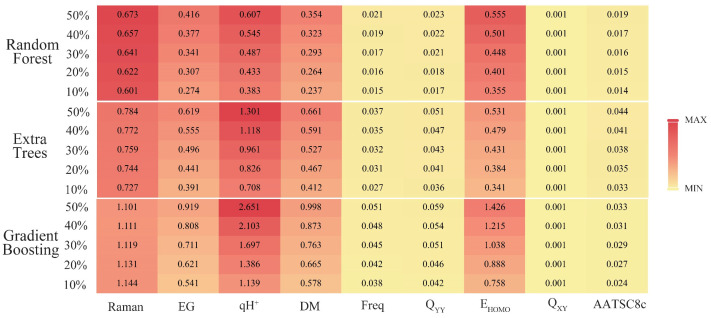
Sensitivity coefficients of key descriptors on bagging-RF, bagging-ERT, and bagging-GBC models.

The absolute value of the sensitivity coefficient under different variation degrees of key descriptors was calculated by Formulas (1)–(3) (Figure 3). As shown in Figure 3, when the degree of key eigenvalues increased, the sensitivity coefficients of all key eigenvalues showed an upward trend, except for the Raman descriptor in the bagging-ERT model. Among the descriptors, the characteristics of Raman, energy gap, qH^+^, dipole moment, and E_HOMO_ were more prominent, and their sensitivity coefficients were all greater than 0.2. However, the characteristics of positive frequency, Q_YY_, Q_XY_, and AATSC8c descriptors were not obvious, and the sensitivity coefficients were all less than 0.05. Therefore, it can be inferred that the key descriptors (i.e., Raman, energy gap, qH^+^, dipole moment, and E_HOMO_) have a more significant impact on the synthesizability of SM derivatives and are in a higher position in the ranking order of the key descriptors’ correlations (Table 6). In contrast, the positive frequencies, Q_YY_, Q_XY_, and AATSC8c had relatively little effect on the synthesizable properties of SM derivatives.

Raman, energy gap, qH^+^, dipole moment, and E_HOMO_ significantly impact the synthesizability of SM derivatives when the change degree of key descriptors is less than 50%. In order to further analyze the changing trend of the synthesizability of SM derivatives when the parameter eigenvalues vary greatly, this paper analyzed the growth rate of the sensitivity coefficients of the characteristic parameters. Thus, the eigenvalues that most significantly affect the synthesizability of SM derivatives were screened, and the growth rates of the sensitivity coefficients of the three models were calculated (Table 7). Results showed that the average growth rates of sensitivity coefficients for the energy gap, qH^+^, dipole moment, and E_HOMO_ were all higher than 10%. However, the average growth rate of Raman’s sensitivity coefficient was less than 3% and even showed a negative growth trend in the bagging-GBC algorithm. After comparison, in the case of significant changes in key eigenvalues, the potential impact of Raman on the molecular synthesis of SM derivatives is much smaller than that of the energy gap, qH^+^, dipole moment, and E_HOMO_. Therefore, energy gap, qH^+^, dipole moment, and E_HOMO_ were the most significant key descriptors affecting the synthesizability of SMs and their derivatives. E_HOMO_ refers to the energy of the highest occupied orbital of a molecule, which is one of the important quantum chemical properties of molecules [38]. The energy gap is the difference in energy between the highest and the lowest occupied orbitals of a molecule. Studies have shown that molecular E_HOMO_ and energy gap values are closely related to molecular stability [39]. The descriptor qH^+^ refers to the maximum charge number of molecular hydrogen ions, and intramolecular hydrogen bonds can enhance the stability of molecules [39]. The dipole moment is the product of the distance between the positive and negative charge centers in a molecule and the charge at the charge center, which is closely related to the effective charge carried by the molecule [40]. Li et al. [24] found that those mentioned above as key characteristic values with significant influence belong to the electrons of the molecule parameter. The molecular structure is associated with key eigenvalues such as E_HOMO_, energy gap, and other properties [41]. Laikov [42] developed a new molecular electronic structure model using the electronic parameters of the molecule and found that molecular structures were inseparable from their electronic parameters. In quantum machine learning, molecular structures and electronic parameters such as E_HOMO_, energy gap, and dipole moment play an extremely important role in studying molecular physico-chemical properties [43]. In summary, the eigenvalues of the molecular electronic descriptors of SM derivatives played an essential role in the training process of ML models. They had a significant impact on the synthetic probability of SM derivatives.

#### 2.4.2. The Mechanism Analysis for the Synthesizability of SMs Derivatives Based on 3D-QSAR Model

In Section 2.4.1, energy gap, qH^+^, dipole moment, and E_HOMO_ were screened out as the key descriptors that significantly affect the synthesis of theoretically designed SM derivatives. In order to verify the above results, the 11 commercially synthesized SMs (positive samples), the top seven SM derivatives with high synthesizability, and the last seven SM derivatives with low synthesizability predicted by the three models were selected. The energy gap, qH^+^, dipole moment, and E_HOMO_ values of the 14 SM derivatives and 11 commercially synthesized SMs are given in Table 8. Compared with the 11 synthesized SMs, the energy gap, qH^+^, dipole moment, and E_HOMO_ eigenvalues of the seven synthesizable SM derivatives changed by 3.72%, −0.66%, −5.70%, and −7.57%, respectively. The energy gap, qH^+^, dipole moment, and E_HOMO_ eigenvalues of the seven none synthesizable SM derivatives changed by 3.72%, −0.66%, −5.70%, and −7.57%, respectively, compared with the 11 commercially synthesized SMs. The results further showed that the eigenvalues of qH^+^, dipole moment, and E_HOMO_ have relatively significant effects on the synthesizable properties of SMs. The higher the value of qH^+^, the larger the value of the dipole moment, and the lower the value of E_HOMO_, the lower the synthesizability of SM derivatives, which is consistent with the conclusion of the sensitivity analysis of the constructed machine learning models.

The 3D-QSAR model can effectively analyze the relationship between molecular structural features and physico-chemical activities [23]. Therefore, the synthesizability of unlabeled samples (SM derivatives) was used as the input to construct an environment-friendly 3D-QSAR prediction model (CoMSIA) for predicting the synthesis of SM derivatives. The data set (n = 35) was composed of a training set (27 SMs) and a test set (9 SMs) for 3D-QSAR model construction and validation, and the template molecule (SM 12) existed in both the training set and test set. The cross-validation coefficient q^2^ is 0.753 > 0.5, indicating that the constructed 3D-QSAR model had a good prediction ability [23]. Relatively high values of non-cross-validation (R^2^ = 0.971 > 0.9 and close to 1.000) and the external test coefficient (r^2^_pred_ = 0.940 > 0.6) further proved the good predictive ability and robustness of the generated models. The standard error of estimate (SEE) of the model was 0.047 < 0.95, which confirmed the good fit ability and predictive ability of the constructed 3D-QSAR model. In addition, in order to verify the rationality of setting the positive samples and unlabeled samples of the machine learning prediction model, this paper also used the constructed 3D-QSAR model to predict the synthesizability of the 11 positive samples (i.e., Phantolide, Celestolide, Tonalid, Galaxolide, Versalide, Musk xylene, Muscone, Musk methy, Musk ambrette, Moskene, and Musk ketone). However, since the 3D-QSAR model can only predict molecules with a common skeleton (Table 9), it cannot predict Muscone without a common benzene ring. Based on the 3D-QSAR model prediction, it was found that 90% of the positive samples had a synthesizability greater than 0.5, indicating that the constructed 3D-QSAR model had high prediction accuracy. At the same time, it showed that the setting of positive and unlabeled samples of SMs and SM derivatives used for machine learning model training is reasonable.

**Figure 4:**
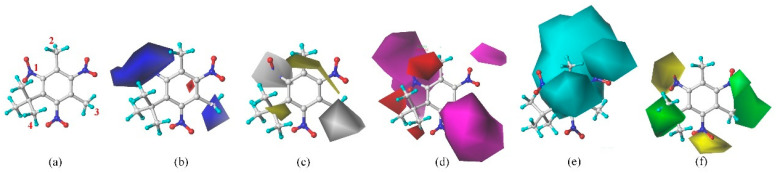
Molecular structure of D50 and the contour maps of 3D-QSAR model. (**a**) Molecular structure of D50; (**b**) electrostatic field; (**c**) hydrophobic field; (**d**) hydrogen bond acceptor field; (**e**) hydrogen bond donor field; (**f**) steric field.

In the contour maps of the CoMSIA model for the molecular synthesizability of environmentally friendly SM derivatives, the contributions of hydrophobic, electrostatic, hydrogen bond acceptor, hydrogen bond donor, and steric fields were 22.6%, 19.8%, 33.2%, 20.4%, and 4.0%, respectively. The results indicated that the hydrophobic, electrostatic, hydrogen bond acceptor, and hydrogen bond donor fields significantly impacted the synthesizability of SM derivatives. In this study, unlabeled sample D50 was taken as a template molecule for analysis (Figure 4). Seven derivatives of MK (i.e., D50, D51, D57, D61, D62, D67, and D76) were selected as examples (Table 10) to analyze the synthesizability mechanism based on the contour maps. Studies showed that increasing the positive electric groups in the blue area in the contour maps was beneficial to chemical activity [22,44]. As shown in Table 10 and Figure 4a, b, compared with the synthesizability of D50 and D51, the non-synthesizable SM derivatives (i.e., D67, D61, D62, D57, and D76) had increased electron-withdrawing groups (-NO_2_) at position 3, which led to the decreased synthesizability of D67, D61, D62, D57, and D76. Previous studies have shown that adding strong electron-withdrawing groups can reduce the E_HOMO_ value of the molecule [45]. Then the energy gap value of the molecule will change significantly, which will increase the charge of some hydrogen atoms in the molecule. According to Long and Niu [46], the higher the qH^+^ value of a molecule, the easier it is to accept electrons and generate electrophilic reactions. The lower the energy gap value of the molecule, the easier it is for nucleophilic and electrophilic reactions to occur, resulting in poor stability (or synthesizability) of the molecule [47,48]. Compared with the synthesizable D50 and D51, the E_HOMO_ values of non-synthesizable SM derivatives (i.e., D57, D61, D62, and D76) were all smaller than D50 and D51. Furthermore, D57, D61, D62, D67, and D76 substitute molecules had lower energy gap values and higher qH^+^ values than D50 and D51. The higher the value of qH^+^, the lower the value of E_HOMO_, and the smaller the energy gap value, the lower the synthesis ability of SMs substitute molecules. It showed that the higher the value of qH^+^, the lower the value of E_HOMO_, and the smaller the energy gap value, the lower the synthesizability of SM derivatives.

Yellow patches appeared surrounding positions 2 and 4 of D50 in the contour map (Figure 4c), indicating that substituting hydrophobic groups in this region are conducive to synthesizing SMs molecules [23]. Compared with the synthesizability of D50, hydrophilic groups (e.g., methoxyl and carboxyl groups) of D67, D61, and D62 were found at position 4. This should be why the synthesizable properties of the D67, D61, and D62 molecules were predicted as “non-synthesizable”. It has been found that the higher the dipole moment value of the molecule, the stronger the polarity of the molecule [49]. Compared with D50, the dipole moment values of the remaining non-synthesizable D67, D61, and D62 were 4.313, 5.726, and 5.915, respectively, which were significantly higher than the dipole moment values of D50 (1.192). Thus, the possible replacement of hydrophilic groups may enhance the polarity of SMs (increased dipole moment value). The change will decrease the synthesis of SMs molecules, which is consistent with the previous conclusions. Thus, the 3D-QSAR model of the molecular synthesizability of environmentally friendly SM derivatives constructed in this paper has a good predictive ability. The results obtained from sensitivity analysis were consistent with those obtained from contour maps: the higher the value of qH^+^, the larger the value of the dipole moment, the lower the value of E_HOMO_, and the smaller the value of the energy gap, the lower the synthesizability of SM molecules.

#### 2.4.3. Mechanism Verification Analysis for the Synthesizability of SM Derivatives Based on Intramolecular Hydrogen Bond Theory

**Figure 5:**
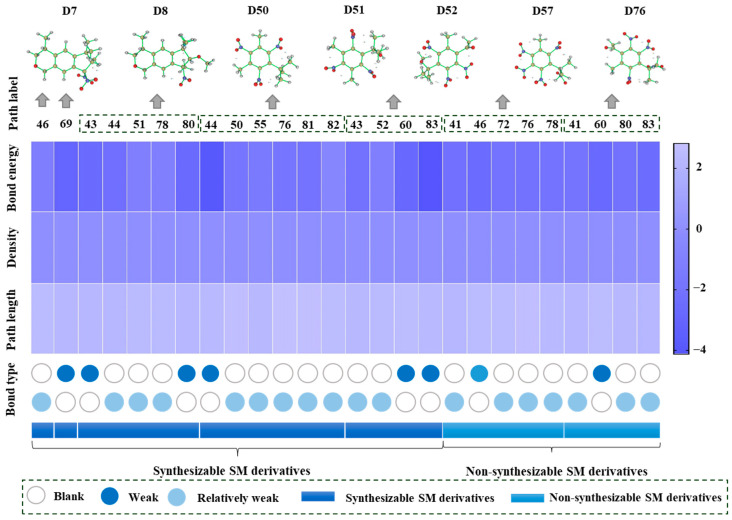
Predicted intramolecular hydrogen bond distribution characteristics of synthesizable and non-synthesizable SM derivatives.

In order to further analyze the synthesizable mechanism of SM derivatives, they were screened based on bagging-RF, bagging-ERT, and bagging-GBC models. The intramolecular hydrogen bond differences of the top seven SM substitute molecules (D7 > D50 > D8 > D25 > D37 > D51 > D52) and the last seven non-synthesizable SM derivatives (D67 < D62 < D39 < D57 < D61 < D76 < D49) were compared to analyze the intrinsic factors affecting the internal reasons of synthesizability. HHCB and MK derivatives from the above 14 SM derivatives were selected as representatives. D7, D8 (synthesizable), and D39, D49 (non-synthesizable) were the derivatives of HHCB. D50, D51, and D52 (synthesizable) and D57 and D76 (non-synthesizable) were the derivatives of MK. For the synthesizable derivatives, D7 contained one intramolecular hydrogen bond C3−H23···O13 (H23···O13 bond length is 2.46 Å), D8 contained one intramolecular hydrogen bond C16–H29···O15 (H29···O15 bond length is 2.22 Å), D50 had five intramolecular hydrogen bonds C10–H28···O17 (H28···O17 bond length is 2.22 Å), C1−H24···O20 (H24···O20 bond length is 2.21 Å), C12−H36···O17 (H36···O17 bond length is 2.42 Å), C12−H35···O14 (H35···O14 bond length is 2.42 Å), C11–H33···O14 (H33···O14 O14 bond length is 2.22 Å), D51 had six intramolecular hydrogen bonds C10–H30···O19 (H30···O19 bond length is 2.36 Å), C13–H35···O22 (H35···O22 bond length is 2.70 Å), C10–H30···O18 (H30···O18 bond length is 2.30 Å), C1–H25···O21 (H25···O21 bond length is 2.82 Å), C9–H27 ···O16 (H27···O16 bond length is 2.73 Å), C8–H31···O16 (H31···O16 bond length is 2.54 Å), and D52 had four intramolecular hydrogen bonds C10−H31·· O20 (H31···O20 bond length is 2.31 Å), C9−H30···O20 (H30···O20 bond length is 2.43 Å), C14–H38···O17 (H38···O17 bond length is 2.49 Å), C12–H34···O23 (the bond length of H34···O23 is 2.34 Å). Among the non-synthesizable SM derivatives, D39 and D49 had no intramolecular hydrogen bonds, while D57 had five intramolecular hydrogen bonds C10–H33··O18 (the bond length of H33···O18 is 2.18 Å), C10–H33···O19 (H33···O19 bond length is 2.48 Å), C1–H28···O21 (H28···O21 bond length is 2.45 Å), C9−H29···O15 (H29···O15 bond length 2.67 Å), C9–H29···O16 (H29···O16 bond length is 2.34 Å), and D76 contained four intramolecular hydrogen bonds C10–H33···O21 (H33···O21 bond length is 2.22 Å), C12–H36···O24 (H36···O24 bond length is 2.51 Å), C9–H30···O19 (H30···O19 bond length is 2.27 Å), C1–H28···O13 (H28···O13 bond length is 2.19 Å) (Table 11). Comparing the number of intramolecular hydrogen bonds, it was found that the formation rate of intramolecular hydrogen bonds in the synthesizable SM derivatives was 100%. The predicted rate of intramolecular hydrogen bond formation in non-synthesizable SM derivatives was only 50% (Figure 5). Since the basic forms of intramolecular hydrogen bonds in the SM derivatives were all C–H···O, it was speculated that the hydrogen bond between the methyl group and the nitro group restricts the rotation of the C–C bond between the benzene ring and the nitro group in the molecular center of the SM derivatives. Thus, D7, D8, D50, D51, D52, D57, and D76 have stable, planar molecular conformations that allow for synthesis. However, the lack of intramolecular hydrogen bonds in D39 and D49 resulted in unclear molecular conformation and unstable structures. Thus, D39 and D49 had low synthesizability. The above inference was consistent with a previous study [50]. The expected molecular conformation and arrangement can be obtained by rationally designing the intramolecular hydrogen bond between the amide and alkoxy groups. The bispyridyl aromatic dicarboxamide derivatives and their complexes can thus be designed [50].

Although the formation rate of intramolecular hydrogen bonds in non-synthesizable SM derivatives was only 50%, D57 and D76 still had intramolecular hydrogen bonds due to structural reasons. Therefore, we further analyzed the influences of intramolecular hydrogen bonds on the synthesizability of MK derivatives (i.e., D50, D51, D52, D57, and D76). Intramolecular hydrogen bond strength is difficult to calculate accurately due to the complex and strict operation process. If the system needs to be cut off and the cutoff point should be saturated, the structure needs to be adjusted to avoid severe steric hindrance. Emamian et al. [51] first proposed that the bond critical point (BCP) electron density can be defined by AIM theory to estimate the intramolecular hydrogen bond strength (E_HB). Emamian et al. [51] also redefined the standard definition of intramolecular hydrogen bond strength, where E_HB > −2.5 kcal/mol is weak strength and E_HB < −2.5 kcal/mol is weak strength. Among these, E_HB > −2.5 kcal/mol is “very weak intensity”, and −14 < E_HB < −2.5 kcal/mol is “weak to medium intensity”. The BCP electron densities of D7, D8, D50, D51, D52, D57, and D76 were calculated by density functional theory (DFT). The strength of the intramolecular hydrogen bonds of these derivatives was then estimated (Table 11, Figure 5). Among the synthesizable SM derivatives, D50 formed three extremely weak intramolecular hydrogen bonds with E_HB of −2.336, −1.102, and −1.101 kcal/mol, respectively, and two weak intramolecular hydrogen bonds with E_HB of −2.704 and −2.707 kcal/mol, respectively. D51 formed five extremely weak intramolecular hydrogen bonds (with the E_HB of −1.960, −1.602, −1.919, −2.100, and −0.677 kcal/mol) and one weak intramolecular hydrogen bond (with E_HB of −4.039 kcal/mol). D52 formed two extremely weak intramolecular hydrogen bonds (E_HB were −2.182, −1.091 kcal/mol) and two weak intramolecular hydrogen bonds (E_HB were −2.885, −4.127 kcal/mol). Among the non-synthesizable SM derivatives, D57 formed four “very weak intensity” intramolecular hydrogen bonds (E_HB were −2.280, −2.105, −2.167, and −2.128 kcal/mol) and one weak intramolecular hydrogen bond (E_HB was −2.576 kcal/mol). D76 formed three “very weak intensity” intramolecular hydrogen bonds (E_HB were −2.049, −2.200, and −2.485 kcal/mol) and one weak intramolecular hydrogen bond (E_HB was −2.781 kcal/mol). Statistical analysis found that the formation rate of weak intramolecular hydrogen bonds in predicted synthesizable SM derivatives was 33.33%, and the weak intramolecular hydrogen bond formation rate in non-synthesized SM derivatives was 22.22%. It showed that “very weak intensity” intramolecular hydrogen bonds are more conducive to synthesizing SM derivatives than weak intramolecular hydrogen bonds. Studies have confirmed that when the intramolecular hydrogen bond is weak, it is mainly dominated by electrostatic interaction forces [51]. This result is consistent with the conclusion in Section 2.4.2 that the electrostatic field can affect the synthesizability of SMs and their derivatives. In addition, based on the sensitivity analysis results of the synthesizability-machine learning model, it can be seen that the energy gap, qH^+^, dipole moment, and E_HOMO_ are key descriptors affecting the synthesizability of SM derivatives. Among them, the size of E_HOMO_ and the energy gap are closely related to molecular stability, and qH^+^ affects the interaction between donor and acceptor in intramolecular hydrogen bonds [39]. Therefore, the intramolecular hydrogen bond strength analysis, the sensitivity analysis of the three synthesizability-machine learning models, and the contour map analysis by the 3D-QSAR model were mutually verified. The higher the intramolecular hydrogen bond strength, electrostatic interaction, qH^+^ value, and energy gap value, the lower the E_HOMO_ value, the more stable the SMs substitute molecule, and the higher the synthesis probability will be.

## 3. Materials and Methods 

### 3.1. Molecular Structures of SMs and SM Derivatives—Literature Review Method

A total of 88 SMs and their derivatives were retrieved, including 11 commercialized SMs (i.e., Phantolide, Celestolide, Tonalid, Galaxolide, Versalide, Musk xylene, Muscone, Musk methy, Musk ambrette, Moskene, and Musk ketone) and 77 environmentally friendly SM derivatives that have been theoretically designed but have not yet been experimentally synthesized. The 77 SM derivatives consist of 19 functionally improved SM derivatives [23], 10 SM derivatives with reduced bioaccumulation ability and enhanced odor sensitivity [24], and 48 SM derivatives with lower abortion risks [18]. The molecular structures of 88 SMs and SM derivatives are shown in Table S1.

### 3.2. Construction of Machine Learning Models for the Synthesizability of SM Derivatives

SMs are widely used in personal care products. Due to the extensive use of SM, environmental and human health problems were accelerated. Therefore, it is of great practical significance to develop and design environmentally friendly SM derivatives. The process of developing SM derivatives takes a long time and much labor. The machine learning (ML) method can reduce unnecessary consumption in the early research stage by helping to discover and terminate the molecular design of SM derivatives with low synthesizability. Therefore, it is necessary to predict the synthesizability of the theoretically designed SM derivatives before synthesizing.

#### 3.2.1. Calculation of Molecular Descriptors of SMs and SM Derivatives—Software Calculation Method

The research found that structural parameters (e.g., Gibbs energy, atomic volume, energy of crystal structure, bond strength, and bond length between adjacent atoms, total energy, atomic energy, formation energy, Bader charge, lattice constant, and electronegativity) can affect the physic-chemical properties of molecules [24,52]. Dolz et al. [53] pointed out that the maximum exfoliation energy is a key descriptor affecting the synthesis of MXenes. Mladenović et al. [54] reported that Highest Occupied Molecular Orbital is one of the key descriptors affecting the synthesis of 4-hydroxy-chromene-2-one. Therefore, the descriptors of SMs molecules were selected in this study as the original eigenvalues of the ML model for predicting the synthesizability of SM derivatives. ChemBioDraw 12.0 (PerkinElmer, USA) was utilized to calculate the physico-chemical parameters, structural parameters, and topological parameters (e.g., critical temperature, critical pressure, hydrophobic constant of organic compounds, Henry constant, heat of formation, steric parameters, molecular weight, and polar surface area) of SMs and SM derivatives [37]. The density functional theory (DFT) in Gaussian 09 software was used to optimize the molecular structures of SMs and SM derivatives at the B3LYP/6-31G basis set level and to calculate the spectral, geometric, and electronic parameters (e.g., Milligan charge, occupied orbital energy, positive frequency value, energy gap value, dipole moment, quadrupole moment, infrared, and Raman spectra) [24]. The topological, electronic, geometric, and physico-chemical parameters (e.g., van der Waals volume and atomic number) of SMs and SM derivatives can be calculated using PaDEL-Descriptor software.

#### 3.2.2. Dimensionality Reduction on Descriptors of SMs and SM Derivatives—Pearson Correlation Coefficient Method

The Pearson correlation coefficient is widely used in statistical analysis, pattern recognition, image processing, and other fields. The Pearson correlation matrix can be used to select suitable descriptors for multiple linear regression analysis [55] and analyze the correlation between production data, dilution attributes, and system efficiency [56]. The molecular descriptors of SMs and SM derivatives calculated in this study may have repeatability. Moreover, in the process of constructing PU machine learning models, not all descriptors can provide different molecular information. Some descriptors are highly correlated and express similar molecular information [56]. Therefore, Pearson’s correlation coefficient method was used in this study to rank the eigenvalues with a high degree of similarity in the correlation coefficient matrix [56,57]. Eliminating features with high similarity and screening out the key descriptors. The Pearson correlation coefficient can be calculated below:

(4) $$r_{xy}=\frac{\sum_{i=1}^{n}\left(x_{i}-\overset{-}{x})(y_{i}-\overset{-}{y}\right)}{\sqrt{\sum_{i=1}^{n}{\left(x_{i}-\overset{-}{x}\right)}^{2}}\sqrt{\sum_{i=1}^{n}{\left(y_{i}-\overset{-}{y}\right)}^{2}}}$$

Among which, $\overset{-}{x}$ and $\overset{-}{y}$ are the average values of the two eigenvalues of SMs and SM derivatives (X and Y), respectively. The absolute value of the Pearson correlation coefficient $r_{xy}$ is less than or equal to 1, indicating a degree of correlation: $r_{xy}$ > 0.5 indicates a strong correlation; 0.3 < $r_{xy}$ < 0.5 indicates a moderate correlation; 0.1 < $r_{xy}$ < 0.3 indicates a weak correlation; and $r_{xy}$ < 0.1 indicates almost no correlation.

SPSS 18.0 software (SPSS Inc., Chicago, IL, USA) is commonly used for Pearson correlation coefficient analysis of eigenvalues. However, since there are over 1000 descriptors of SMs and SM derivatives in this study, the Pearson correlation coefficient was calculated and analyzed by self-writing “code packages” in python software [28]. The correlation between each molecular descriptor was calculated as well. The number of eigenvalues required for PU machine learning was set by adjusting the classification threshold (which was set to *p* = 0.6 in this study), and the highly correlated descriptors were eliminated independently. The specific process code is shown in Figure S1.

#### 3.2.3. Molecular Synthesizable Prediction Model for SM Derivatives—Bagging-Random Forests Algorithm

In this paper, the number of positive samples (i.e., 11 synthesized and commercialized SMs) used to construct the PU machine learning prediction model of synthesizability is small, and its number is much smaller than that of unlabeled samples (i.e., 77 theoretically designed SM derivatives). Thus, traditional classifiers such as the random forest (RF) method are more appropriate. A bagging classification model was constructed in this study based on the basic classifier (i.e., RF) and the independent variables (i.e., descriptors obtained by dimensionality reduction through the Pearson correlation coefficient method) [58].

In the Bagging-RF classification method, the synthesized commercial SMs were set as positive samples (marked as “1”), and the theoretically designed yet unsynthesized environment-friendly SM derivatives were set as unlabeled samples (marked as “0”). The above samples were used to construct a PU machine learning classification model. During the model training process, unlabeled samples are divided into unlabeled synthesizable samples and unlabeled non-synthesizable samples, and their synthesizability can be output simultaneously. By adjusting the n_estimators, criterion, and random_state parameters inside the RF algorithm and the n_estimators, max_samples, random_state, and other parameters in the PU classifier, a PU classification model with a model evaluation score (out-of-bag (OOB) score) greater than 0.7 is obtained. Finally, the model with a high oob_score (greater than 0.7) is used to predict the synthesizability of the positive and unlabeled samples. In this way, the synthesizability of SM derivatives was obtained. When the synthesizability is >0.5, it is considered synthesizable, and when the synthesizability is <0.5, it is considered not synthesizable [28]. The specific code package is shown in Figure S2.

#### 3.2.4. Molecular Synthesizable Prediction Model for SM Derivatives—Bagging-Extremely Randomized Tree Classifier Algorithm

In this paper, the bagging-Extremely Randomized Tree Classifier (ERT) machine learning method was also used to predict the synthesizability of environmentally friendly SM derivatives. ERT was used as an alternative algorithm for PU machine learning predictive models to demonstrate that the synthesis of SM derivatives is not dependent on a specific predictive model. The ERT algorithm is a machine learning method based on tree structure for decision-making. Its algorithm is very similar to the random forest algorithm, which is composed of many decision trees. The RF uses randomly selected samples. In comparison, the ERT uses all samples with randomly selected molecular characteristics because the split is random. Therefore, to some extent, ERT is more appropriate than the prediction results simulated by the RF algorithm [59]. The RF model selects the optimal forked features in a feature subset, while the ERT model randomly selects the forked features [60]. The ERT model can adjust the minority class of target features in classification by reducing the variance of tree-splitting nodes [60]. The ERT model was used to reduce the variance inherent in many tree-based and neural network algorithms through an enhanced tree splitting technique. Due to its randomization properties for numerical inputs, ERT was very efficient in solving problems involving a large number of numerical features [60]. Thus, this paper adopted the ERT model for the synthesizability prediction of unlabeled samples. An ensemble of decision trees generated a decision function. The classifier took an input feature vector and classified it for each tree in a forest-like structure. Then the labeled class was output based on the majority vote [61]. The specific code package is shown in Figure S3.

ERT is an extension of RF, where a further randomization stage is added for selecting cut points and, at the same time, randomizes the attributes in RF, randomly selecting attributes and splitting cut points [62]. According to Soltaninejad et al. [62], each tree was determined by $t\epsilon \left\{1\ldots T\right\}$, where *T* is the number of random trees. For a given data point *x* and data set Dtrain, the feature vector is represented by f Dtrain, and the feature vector is represented by $f\left(x,D_{train}\right)$. To classify the data from class *c*, each tree learned a weak predictor $pt\left(c|f\left(D_{train}\right)\right)$ for an n-dimensional feature representation. During testing, for an unseen data point *x*’, the probability of belonging to class c is calculated by the average of the probabilities on all trees, as shown in Equation (5):

(5) $$p\left(c|f\left(x^{\prime },D\right)\right)=\frac{1}{T}\sum_{t-1}^{T}p_{t}\left(c|f\left(x^{\prime },D\right)\right)$$

#### 3.2.5. Molecular Synthesizable Prediction Model for SM Derivatives—Bagging-Gradient Boosting Classifier

In this paper, the machine learning of the bagging-gradient boosting classifier (GBC) was used to construct and compare the predictive models for the synthesis of SM derivatives to prove once again that the synthetic properties of SM derivatives do not depend on specific prediction models. GBC is an ensemble classifier that performs well when the number of variables exceeds the number of samples (high-dimensional data) [63]. GBC has been applied to the status classification of water quality. Experiments showed that GBC was more effective in classifying water quality status than the AdaBoost classifier, supporting vector classifiers, and random forest classifiers [64]. Gradient boosting is a method used to develop classification and regression models to optimize the learning process of the models, which are mostly nonlinear and are more broadly known as decision trees or regression trees. The GBC algorithm [65,66] first used prior information to initialize the classifier $F_{0}\left(x\right)$. $F_{0}\left(x\right)$ is the average value of the training target value, and the proportion of *y* = 1 in the training sample is $P\left(Y=1|x\right)$. For the first iteration, *t* = 1, the formula was shown as follows:

(6) $$F_{0}\left(x\right)=\log\left(\frac{P\left(Y=\left.1\right|x\right)}{1-P\left(Y=1|x\right)}\right)$$

The loss function negative gradient $r_{m,i}$ can be calculated by Formula (7), among which *m* = 1, 2, 3, …, *M*.

(7) $$r_{m,i}={\left[\frac{\partial L\left(y_{i},F\left(x_{i}\right)\right)}{\partial F\left(x\right)}\right]}_{F\left(x\right)=F_{m-1}\left(x\right)}=y_{i}-\frac{1}{1+e^{-F\left(x_{i}\right)}}$$

Best-fit value $c_{m,j}$ can be calculated using Formula (8). The regression tree was used to fit the data $\left(x_{i},r_{m,j}\right)$, among which *i* = 1, 2, 3, …, *N*. The leaf nodes under the *m*th regression tree are $R_{m,j}$, among which $j=1,2,3\cdots ,J_{m,}$ $J_{m}$ is the number of leaf nodes for the mth regression tree.

(8) $$c_{m,j}=\frac{\sum_{x_{i\in }R_{m,j}}r_{m,i}}{\sum_{x_{i\in }R_{m,j}}(y-r_{m,i})\left(1-y+r_{m,i}\right)}$$

The calculation of $F_{m}(x)$ classifier:

(9) $$F_{m}\left(x\right)=F_{m-1}\left(x\right)+\sum_{j=1}^{J_{m}}c_{m,j}I\left(x\in R_{m,j}\right)$$

The best classifier $F_{M}(x)$:

(10) $$F_{M}\left(x\right)=F_{0}\left(x\right)+\sum_{m=1}^{M}\sum_{j=1}^{J_{m}}c_{m,j}I\left(x\in R_{m,j}\right)$$

The final computational form of the classifier model:

(11) $$P\left(Y=1|x\right)=\frac{1}{1+e^{-F_{M}(x)}}$$

The detailed code package is provided in Figure S4.

### 3.3. Mechanism Analysis of the Synthesizability of SM Derivatives

#### 3.3.1. Identification and Analysis of Key Descriptors Affecting the Synthesis of SM Derivatives—2D-QSAR Model

Sensitivity analysis is mainly used to analyze the sensitivity of model output values to changes in characteristic parameters [29]. In this study, the linear regression module of SPSS 18.0 software (SPSS Inc., Chicago, IL, USA) was used to construct two-dimensional quantitative structure-activity relationships (2D-QSAR) models. The models were constructed based on the dependent variable (i.e., synthesizability of SM derivatives) and the independent variables (i.e., key descriptors screened by the machine learning model). The structure-activity relationship between the synthesizability of SM derivatives and the characteristic parameters was thus created. Sensitivity analysis was then carried out with the help of characteristic parameter coefficients to screen the key descriptors, which helps to accurately analyze the influence of key descriptors on the synthesizability of SM derivatives [30].

The sensitivity coefficient can be calculated as follows:

(12) $$SC_{i}=\left(\Delta Y_{i}/Y_{i}\right)/\left(\Delta X_{i}/X_{i}\right)$$

where *SC_i_* represents the sensitivity coefficient of the input feature parameter *i*, Δ*X_i_*/*X_i_* indicates the change rate of the input feature parameters, Δ*Y_i_*/*Y_i_* is the synthesizability change rate of SM derivatives. The sensitivity analysis on each descriptor was conducted with the help of each descriptor’s coefficient in the linear regression model. The sensitivity coefficients for each descriptor varying by 10%, 20%, 30%, 40%, and 50% of its value were calculated, which helped to screen out the key descriptors.

#### 3.3.2. Molecular Force Field Analysis Affecting Molecular Synthesizability of SM Derivatives—3D-QSAR Model

The chemical structures (i.e., designed SM derivatives) and their synthesizability were used as independent and dependent variables, respectively, for the establishment of 3D-QSAR models. The synthesizability of unlabeled samples (i.e., SM derivatives) is predicted by the ML model with the highest model evaluation score. Using the SM derivative with the highest synthesizability as a template, a 3D-QSAR model about the synthesizability of environmentally friendly SM derivatives was constructed. The molecules of SM derivatives were optimized using the Tripos force field and Gasteiger-Hückel charges [67]. The Powell method was optimized up to 10,000 times, the energy convergence gradient value was set to 0.005 kJ/mol, and the rest of the parameters were set to default values [5]. The template molecules were then aligned with other SMs using the align database command in SYBYL.

The constructed 3D-QSAR model was then used to predict the synthesizability of positive samples (i.e., synthesized and commercialized) for verifying the molecular synthesizability of SMs and their derivatives predicted by the PU ML model. The plausibility of the positive and unlabeled samples used in the PU ML model was also verified. At the same time, the coupling of the force field information of the 3D-QSAR contour maps with the key descriptors affecting the molecular synthesis of SM derivatives was used to analyze the synthesizable mechanism of SM derivatives further.

#### 3.3.3. Theoretical Analysis of Intramolecular Hydrogen Bonds Affecting Synthesizability of SM Derivatives—DFT Method

The stability of chemicals is closely related to the ease of synthesis [68], and intramolecular hydrogen bonds can directly affect the stability of compound molecules [32]. For example, Berl et al. [69] reported in “Nature” that the intramolecular hydrogen bond between the amide group and the pyridine group could ensure that the pyridine carboxamide oligomer has a stable double helix structure. Intramolecular hydrogen bonds have directional non-covalent interactions. According to this interaction, highly planar molecular conformations can be designed, and finally, newly designed compounds with high stability and easy synthesis can be obtained [33]. The basic form of intramolecular hydrogen bonds is D−H···A, where D is a hydrogen bond donor and A is a hydrogen bond acceptor, and the length of H···A is usually less than 3.2 Å [70]. This paper used the DFT method in Gaussian 09 software (Gaussian, Inc., Wallingford, CT, USA) to optimize the molecular structure of SM derivatives under the B3LYP/6-31G basis set [71]. The bond critical point (BCP) was obtained by using the topology analysis module of the wave function analysis program Multiwfn, and finally, the intramolecular hydrogen bond strength (E_HB) was estimated based on the BCP electron density defined by the Atoms in Molecules (AIM) theory. The AIM theory is mainly based on the topological properties of the electron density function to describe the bonding situation in the SM derivative molecules, where the point between two interacting atoms in the SM substitute molecule is defined as BCP [4].

## 4. Conclusions

This study investigated the synthesizability of environmentally friendly SM derivatives by integrating machine learning, 3D-QSAR, 2D-QSAR, and DFT methods. Seven of the 77 SM derivatives screened were found to have high synthesizability and low environmental risks. SM derivatives were recommended based on their performances. D37 and D52, with low abortion risks, can be used as alternatives to HHCB and MK. D52, with improved odor sensitivity (increased 10.77%), can be used as an alternative with improved functional properties. From the perspective of reducing the dermal exposure risks of SMs, D25 had a lower skin keratin enrichment (−17.25) and thus could be used as an alternative to PHAN. In addition, the synthesizability of SM derivatives was analyzed by 3D-QSAR, 2D-QSAR, and DFT methods. Based on the mechanism analysis, descriptors such as qH^+^ and energy gap can influence the synthesizability of SMs and their derivatives. This study analyzed the molecular structure and physico-chemical parameters of SM derivatives to accurately screen high-performance, environmentally friendly SM derivatives with high synthesizability. This research has remarkably improved in synthesizing high-performance alternatives for emerging contaminants from molecular aspects. This technology can also be applied to other fields, promoting the discovery and development of more advanced functional materials in various areas.

## Figures and Tables

**Table 1:** Synthesizability of SM derivatives calculated through bagging-RF algorithm.

SM Derivatives	Synthesizability	SM Derivatives	Synthesizability	SM Derivatives	Synthesizability
D1	0.33	D27	0.47	D53	0.32
D2	0.41	D28	0.55	D54	0.39
D3	0.52	D29	0.43	D55	0.34
D4	0.46	D30	0.36	D56	0.46
D5	0.51	D31	0.42	D57	0.25
D6	0.49	D32	0.43	D58	0.31
D7	0.65	D33	0.57	D59	0.49
D8	0.61	D34	0.50	D60	0.50
D9	0.27	D35	0.55	D61	0.23
D10	0.45	D36	0.38	D62	0.21
D11	0.46	D37	0.62	D63	0.45
D12	0.43	D38	0.55	D64	0.36
D13	0.42	D39	0.25	D65	0.33
D14	0.26	D40	0.27	D66	0.41
D15	0.48	D41	0.35	D67	0.20
D16	0.42	D42	0.33	D68	0.25
D17	0.21	D43	0.39	D69	0.40
D18	0.29	D44	0.36	D70	0.48
D19	0.35	D45	0.34	D71	0.31
D20	0.30	D46	0.38	D72	0.23
D21	0.36	D47	0.45	D73	0.33
D22	0.57	D48	0.37	D74	0.30
D23	0.44	D49	0.25	D75	0.24
D24	0.45	D50	0.62	D76	0.29
D25	0.63	D51	0.57	D77	0.43
D26	0.37	D52	0.50		

**Table 2:** Synthesizability of SM derivatives calculated through the bagging-ERT algorithm.

SM Derivatives	Synthesizability	SM Derivatives	Synthesizability	SM Derivatives	Synthesizability
D1	0.37	D27	0.38	D53	0.44
D2	0.37	D28	0.52	D54	0.44
D3	0.48	D29	0.47	D55	0.40
D4	0.51	D30	0.19	D56	0.54
D5	0.49	D31	0.25	D57	0.26
D6	0.44	D32	0.42	D58	0.34
D7	0.65	D33	0.71	D59	0.46
D8	0.58	D34	0.52	D60	0.40
D9	0.19	D35	0.48	D61	0.16
D10	0.59	D36	0.29	D62	0.24
D11	0.36	D37	0.56	D63	0.50
D12	0.37	D38	0.42	D64	0.37
D13	0.35	D39	0.15	D65	0.22
D14	0.23	D40	0.22	D66	0.28
D15	0.37	D41	0.25	D67	0.14
D16	0.37	D42	0.30	D68	0.31
D17	0.27	D43	0.36	D69	0.31
D18	0.24	D44	0.32	D70	0.45
D19	0.32	D45	0.23	D71	0.29
D20	0.22	D46	0.27	D72	0.14
D21	0.35	D47	0.32	D73	0.29
D22	0.52	D48	0.31	D74	0.29
D23	0.50	D49	0.21	D75	0.28
D24	0.28	D50	0.79	D76	0.27
D25	0.50	D51	0.60	D77	0.33
D26	0.25	D52	0.56		

**Table 3:** Synthesizability of SM derivatives calculated through bagging-GBC.

SM Derivatives	Synthesizability	SM Derivatives	Synthesizability	SM Derivatives	Synthesizability
D1	0.32	D27	0.30	D53	0.08
D2	0.47	D28	0.44	D54	0.31
D3	0.42	D29	0.49	D55	0.09
D4	0.30	D30	0.05	D56	0.20
D5	0.47	D31	0.26	D57	0.06
D6	0.63	D32	0.50	D58	0.06
D7	0.77	D33	0.49	D59	0.47
D8	0.61	D34	0.49	D60	0.29
D9	0.14	D35	0.57	D61	0.06
D10	0.49	D36	0.13	D62	0.04
D11	0.29	D37	0.57	D63	0.16
D12	0.36	D38	0.25	D64	0.14
D13	0.30	D39	0.05	D65	0.07
D14	0.13	D40	0.10	D66	0.15
D15	0.32	D41	0.10	D67	0.03
D16	0.17	D42	0.15	D68	0.11
D17	0.02	D43	0.26	D69	0.07
D18	0.14	D44	0.13	D70	0.34
D19	0.25	D45	0.05	D71	0.05
D20	0.11	D46	0.11	D72	0.03
D21	0.19	D47	0.25	D73	0.06
D22	0.34	D48	0.20	D74	0.05
D23	0.26	D49	0.08	D75	0.09
D24	0.18	D50	0.70	D76	0.06
D25	0.61	D51	0.51	D77	0.22
D26	0.06	D52	0.50		

**Table 4:** Prediction of the top 10 SM derivatives for synthesizability predicted by bagging-GBC, bagging-ERT, and bagging-RF models.

Ranking	SM Derivatives	Predicted Synthesizability by Bagging-RF	SM Derivatives	Predicted Synthesizability by Bagging-ERT	SM Derivatives	Predicted Synthesizability by Bagging-GBC
1	D7	0.65	D50	0.79	D7	0.77
2	D25	0.63	D33	0.71	D50	0.70
3	D37	0.62	D7	0.65	D6	0.63
4	D50	0.62	D51	0.60	D8	0.61
5	D8	0.61	D10	0.59	D25	0.61
6	D22	0.57	D8	0.58	D35	0.57
7	D51	0.57	D37	0.56	D37	0.57
8	D33	0.57	D52	0.56	D51	0.51
9	D38	0.55	D56	0.54	D52	0.50
10	D28	0.55	D34	0.52	D32	0.50

**Table 5:** Environmental risk and functional properties of seven selected SM derivatives.

Name	Abortion Risk (Predicted)				Biotoxicity (Predicted)		Bioaccumulation Ability (Predicted)		Functional Property (Odor Sensitivity, Predicted)	
SMs and SM Derivatives	Docked to 1A52	Change Rates	Docked to 1A28	Change Rates	LC_50_ mg/L (fish)	Change rates	Docked to 4ZRY	Change Rates	Docked to OR5AN1	Change Rates
HHCB	1.93 **		1.958 **		0.032 ^		120.398		111.766	
D7	1.883 **	−2.42%	1.962 **	0.20%	8.387	26,109.38%	116.022	−3.63%	116.639	4.36%
D8	1.858 **	−3.72%	1.962 **	0.20%	2.540	7837.50%	108.7	−9.72%	101.292	−9.37%
D37	1.869	−3.15%	1.934	−1.23%	0.154 ^	381.25%	113.439	−5.78%	119.79	7.18%
MK	1.719 **		1.855 **		0.242		114.902		105.461	
D50	1.751 **	1.86%	1.833 **	−1.19%	0.198	−18.18%	109.97	−4.29%	114.814	8.87%
D51	1.826 **	6.22%	1.804 **	−2.75%	1.477	510.33%	99.259	−13.61%	108.348	2.74%
D52	1.687 **	−1.86%	1.839 **	−0.86%	6.214	2467.77%	119.211	3.75%	116.82	10.77%
PHAN	1.903 **		1.951 **		0.069		89.31		139.604	
D25	1.917	0.79%	1.95	−0.04%	0.582	743.48%	73.57 *	−17.25%	150.283 *	7.65%

* Data obtained from [24]; ** Data obtained from [18]; ^ Data obtained from [23].

**Table 6:** Correlation ranking of 16 key descriptors in bagging-RF, bagging-ERT, and bagging-GBC models.

Importance Ranking of Key Descriptors	PU Machine Learning Methods		
	Bagging-RF Method	Bagging-ERT Method	Bagging-GBC Method
1	Raman	Raman	Raman
2	Energy gap	qH^+^	qH^+^
3	qH^+^	Energy gap	Energy gap
4	Dipole moment	Dipole moment	Dipole moment
5	Positive frequency	Q_YY_	Positive frequency
6	E_HOMO_	Positive frequency	E_HOMO_
7	Q_YY_	E_HOMO_	AATSC8c
8	Q_XY_	Q_XY_	Q_YY_
9	Q_XZ_	AATSC8c	Q_XZ_
10	AATSC8c	GATS3s	Q_YZ_
11	Q_YZ_	GATS5c	GE
12	GATS3s	Q_XZ_	GATS5c
13	GATS5c	Q_ZZ_	Q_XY_
14	GATS6c	GE	GATS6c
15	GE	GATS6c	Q_ZZ_
16	Q_ZZ_	Q_YZ_	GATS3s

**Table 7:** Growth rate of the key descriptors with high correlation in the bagging-RF, bagging-ERT, and bagging-GBC models.

ML Methods	Key Descriptors	Parameter Growth Rate of the Descriptor				
		10–20%	20–30%	30–40%	40–50%	Average
Bagging-RF	Raman	3.44%	3.00%	2.64%	2.34%	2.85%
	Energy gap	11.88%	11.18%	10.60%	10.11%	10.94%
	qH^+^	13.03%	12.39%	11.88%	11.48%	12.20%
	Dipole moment	11.49%	10.77%	10.17%	9.67%	10.53%
	E_HOMO_	12.73%	12.07%	11.54%	11.11%	11.86%
Bagging-ERT	Raman	2.33%	2.01%	1.75%	1.54%	1.91%
	Energy gap	13.10%	12.46%	11.96%	11.57%	12.27%
	qH^+^	16.60%	16.34%	16.29%	16.44%	16.42%
	Dipole moment	13.34%	12.72%	12.24%	11.87%	12.54%
	E_HOMO_	12.58%	11.92%	11.38%	10.94%	11.70%
Bagging-GBC	Raman	−1.18%	−0.99%	−0.84%	−0.72%	−0.94%
	Energy gap	14.74%	14.25%	13.91%	13.71%	14.15%
	qH^+^	21.69%	22.48%	23.87%	26.08%	23.53%
	Dipole moment	15.14%	14.69%	14.41%	14.27%	14.63%
	E_HOMO_	17.16%	16.99%	17.04%	17.33%	17.13%

**Table 8:** Four key descriptors of positive samples, synthesizable, and non-synthesizable SM derivatives on three models.

Synthesizability	Name	Energy Gap	qH^+^	Dipole Moment	E_HOMO_
Synthesizable SM derivatives	D7	0.174	0.173	2.279	−0.239
	D8	0.164	0.178	5.101	−0.252
	D25	0.184	0.184	2.594	−0.241
	D37	0.222	0.162	2.827	−0.226
	D50	0.178	0.213	1.192	−0.284
	D51	0.173	0.213	3.018	−0.281
	D52	0.161	0.210	4.471	−0.279
Average		0.180	0.191	3.069	−0.257
Non-synthesizable SM derivatives	D39	0.168	0.410	2.679	−0.241
	D49	0.222	0.394	2.892	−0.215
	D57	0.178	0.221	4.548	−0.311
	D61	0.158	0.431	4.313	−0.294
	D62	0.160	0.217	5.726	−0.283
	D67	0.139	0.415	5.915	−0.279
	D76	0.160	0.222	5.055	−0.288
Average		0.169	0.330	4.447	−0.273
Positive samples	PHAN	0.186	0.175	3.458	−0.232
	ABDI	0.184	0.181	3.213	−0.231
	AHTN	0.187	0.181	3.457	−0.233
	HHCB	0.220	0.164	1.551	−0.221
	VER	0.190	0.183	3.040	−0.234
	MA	0.162	0.212	4.276	−0.258
	MC	0.229	0.185	2.161	−0.241
	MK	0.173	0.193	3.034	−0.267
	MMP	0.169	0.195	1.542	−0.169
	MUSCONE	0.175	0.206	4.471	−0.269
	MX	0.178	0.213	1.192	−0.284
Average		0.187	0.190	2.854	−0.240

**Table 9:** The predicted synthesizability of positive samples using 3D-QSAR model.

SMs	Molecular Structures	Synthesizability	SMs	Molecular Structures	Synthesizability
Phantolide	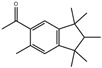	0.589	Muscone	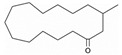	N/A
Celestolide	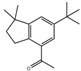	0.631	Musk methy	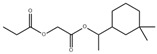	0.627
Tonalid	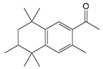	0.582	Musk ambrette	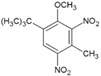	0.494
Galaxolide	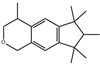	0.586	Moskene	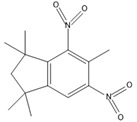	0.363
Versalide	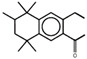	0.661	Musk ketone	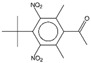	0.731
Musk xylene	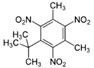	0.544			

**Table 10:** Synthesizable and non-synthesizable musk ketone (MK) derivatives.

Synthesizability	MK Derivatives	Synthesizability Predicted by Bagging-RF	Synthesizability Predicted by Bagging-ERT	Synthesizability Predicted by Bagging-GBC	Molecular Structures
Synthesizable	D50	0.62	0.79	0.70	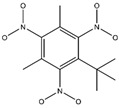
	D51	0.57	0.60	0.51	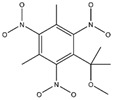
Non-synthesizable	D57	0.25	0.26	0.06	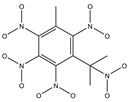
	D61	0.23	0.16	0.06	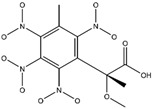
	D62	0.21	0.24	0.04	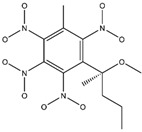
	D67	0.20	0.14	0.03	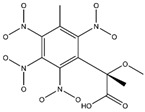
	D76	0.29	0.27	0.06	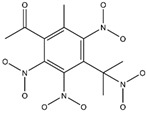

**Table 11:** The intramolecular hydrogen bond analysis of predicted synthesizable and non-synthesizable SM derivatives.

Predicted Synthesizability	SM Derivatives	Intramolecular Hydrogen Bonding Paths		Bond Length (Å)	Electron Density (a.u.)	Hydrogen Bond Strength (kcal/mol)	Hydrogen Bond Type
Synthesizable	D7	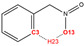	C3–H23···O13	2.460	0.009	−1.237	Very week
	D8	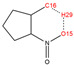	C16–H29···O15	2.220	0.017	−3.130	Week
	D50	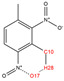	C10–H28···O17	2.220	0.015	−2.704	Week
		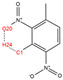	C1–H24···O20	2.210	0.014	−2.336	Very week
		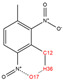	C12–H36···O17	2.420	0.008	−1.102	Very week
		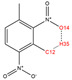	C12–H35···O14	2.420	0.008	−1.101	Very week
		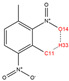	C11–H33···O14	2.220	0.015	−2.707	Week
	D51	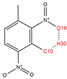	C10–H30···O19	2.360	0.021	−4.039	Week
		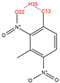	C13–H35···O22	2.700	0.012	−1.960	Very week
		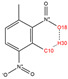	C10–H30···O18	2.300	0.011	−1.602	Very week
		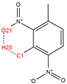	C1–H25···O21	2.820	0.012	−1.919	Very week
		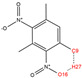	C9–H27···O16	2.730	0.013	−2.100	Very week
		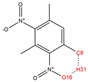	C8−H31···O16	2.540	0.006	−0.677	Very week
	D52	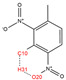	C10−H31···O20	2.310	0.013	−2.182	Very week
		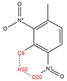	C9−H30···O20	2.430	0.008	−1.091	Very week
		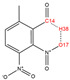	C14−H38···O17	2.490	0.016	−2.885	Week
		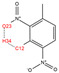	C12−H34···O23	2.340	0.022	−4.127	Week
Non-synthesizable	D39	-	-	-	-	-	-
	D49	-	-	-	-	-	-
	D57	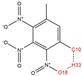	C10−H33···O18	2.180	0.014	−2.280	Very week
		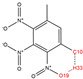	C10−H33···O19	2.480	0.015	−2.576	Week
		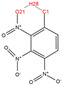	C1−H28···O21	2.450	0.013	−2.105	Very week
		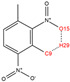	C9−H29···O15	2.670	0.013	−2.167	Very week
		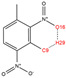	C9−H29···O16	2.340	0.013	−2.128	Very week
	D76	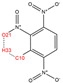	C10−H33···O21	2.220	0.013	−2.049	Very week
		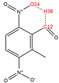	C12−H36···O24	2.510	0.016	−2.781	Week
		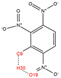	C9−H30···O19	2.270	0.013	−2.200	Very week
		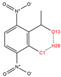	C1–H28···O13	2.190	0.014	−2.485	Very week

## Data Availability

Data will be provided upon request.

## Supplementary Materials

The following supporting information can be downloaded at: https://www.mdpi.com/article/10.3390/ijms24032768/s1.
