# Supplementary material for: Synthesis of Synthetic Musks: A Theoretical Study Based on the Relationships between Structure and Properties at Molecular Scale
Source: Int J Mol Sci. 2023 Feb 1;24(3):2768. doi: 10.3390/ijms24032768 (PMC9917709; doi:10.3390/ijms24032768)
Supplement: Supplementary file 1 [file ijms-24-02768-s001.zip › Supplementary I.pdf]

Supplement Materials

Synthesis of Synthetic Musks: A Theoretical Study Based on the Relationships between Structure and Properties at Molecular Scale

Xixi Li [1,2,3,†], Hao Yang [4,†], Yuanyuan Zhao [4], Qikun Pu [4], Tingzhi Xu [4], Rui Li [1,2,*] and Yu Li [4,*]

[1] State Key Laboratory of Environmental Criteria and Risk Assessment, Chinese Research Academy of Environmental Sciences, Beijing 100012, China

[2] State Environmental Protection Key Laboratory of Ecological Effect and Risk Assessment of Chemicals, Chi-nese Research Academy of Environmental Sciences, Beijing 100012, China

[3] Northern Region Persistent Organic Pollution Control (NRPOP) Laboratory, Faculty of Engineering and Ap-plied Science, Memorial University, St. John's, NL A1B 3X5, Canada

[4] MOE Key Laboratory of Resources and Environmental Systems Optimization, North China Electric Power University, Beijing 102206, China

[*] Correspondence: lirui@craes.org.cn (R.L.); liyuxx@ncepu.edu.cn (Y.L.)

[†] These authors contributed equally to this work.

Table S1 Molecular structures of 88 SMs and SMs derivatives

Name	Molecular structures	Name	Molecular structures
D1	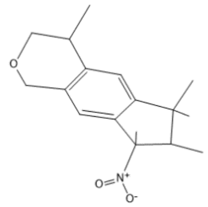	D45	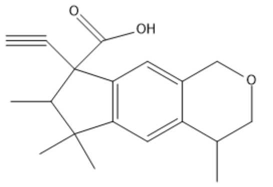
D2	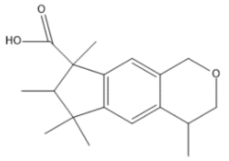	D46	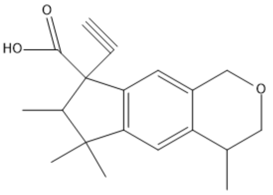
D3	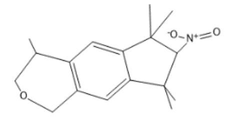	D47	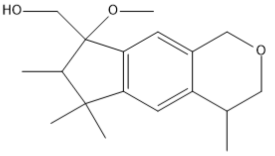
D4	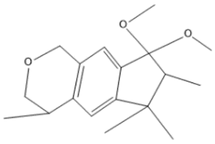	D48	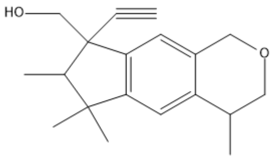
D5	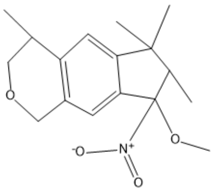	D49	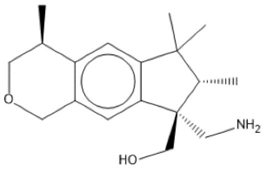
D6	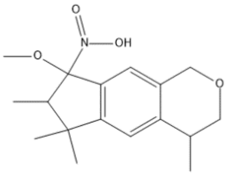	D50	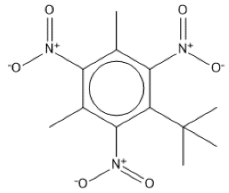
D7	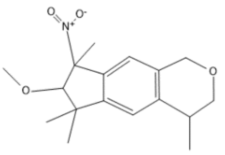	D51	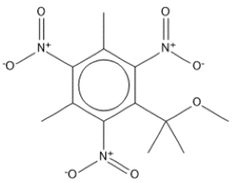
D8	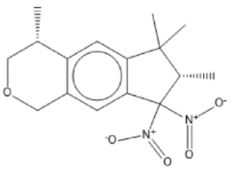	D52	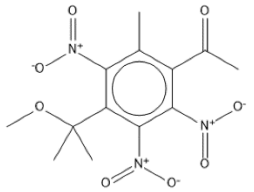

D9	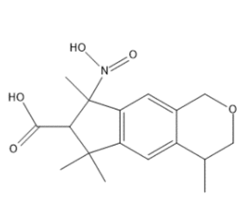	D53	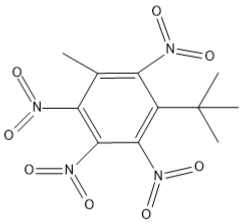
D10	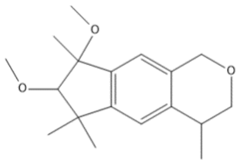	D54	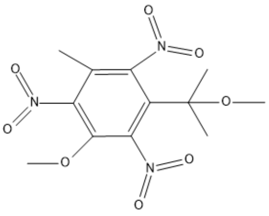
D11	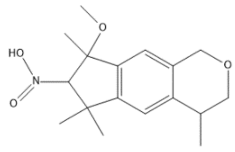	D55	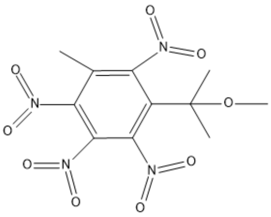
D12	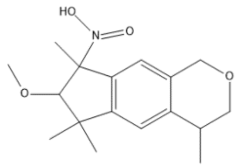	D56	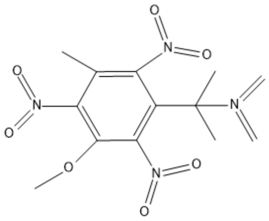
D13	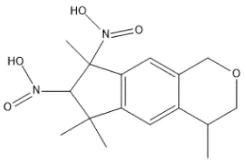	D57	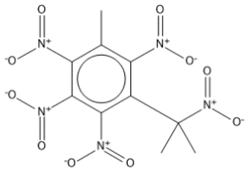
D14	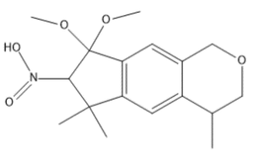	D58	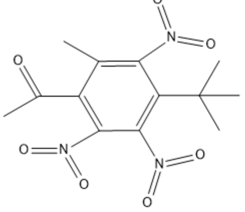
D15	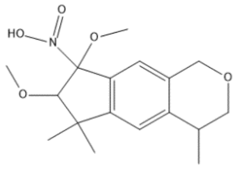	D59	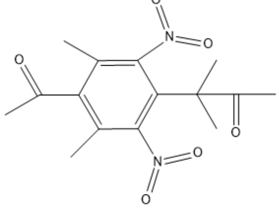

D16	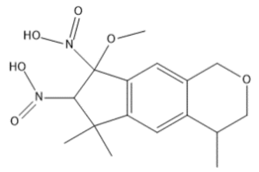	D60	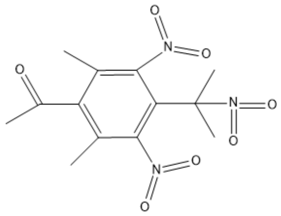
D17	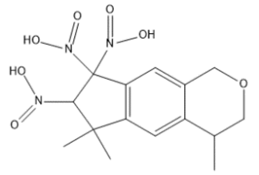	D61	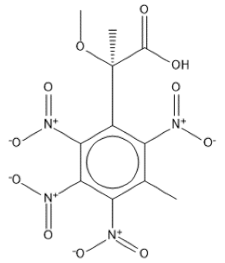
D18	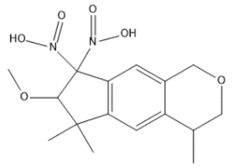	D62	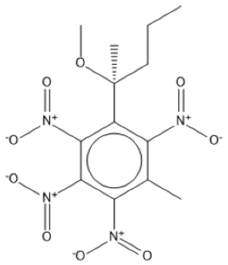
D19	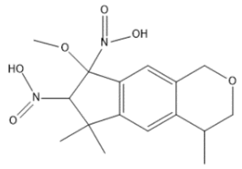	D63	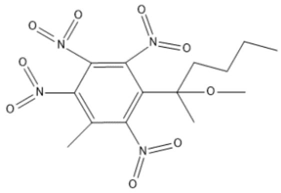
D20	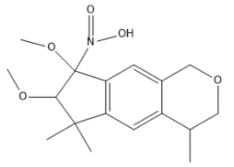	D64	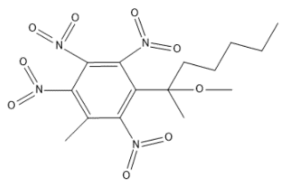
D21	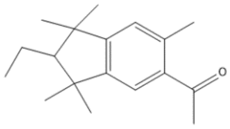	D65	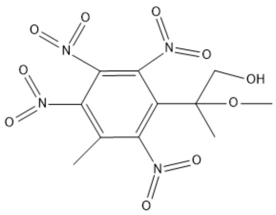
D22	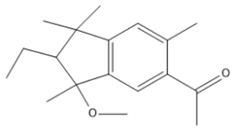	D66	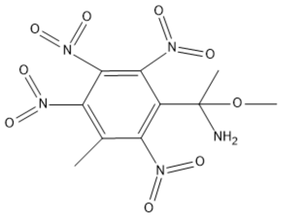

D23	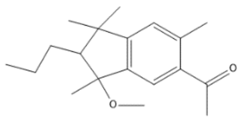	D67	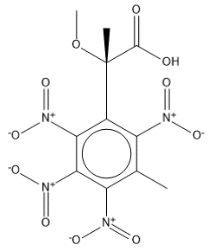
D24	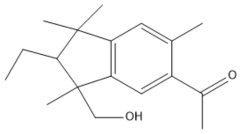	D68	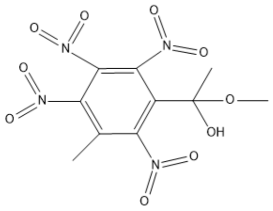
D25	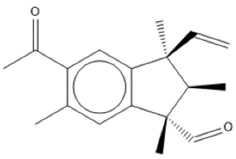	D69	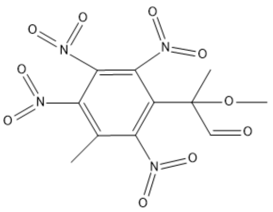
D26	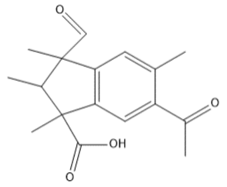	D70	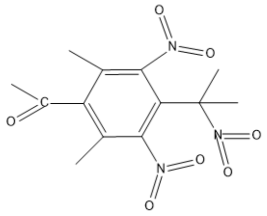
D27	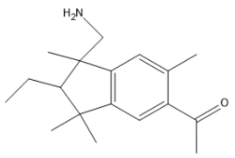	D71	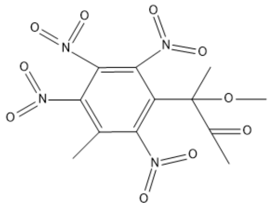
D28	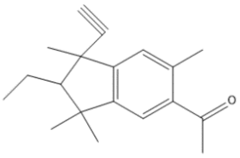	D72	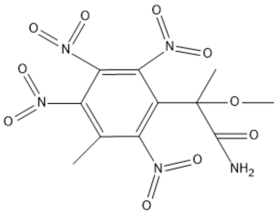
D29	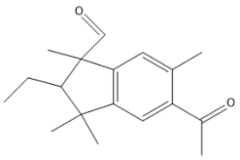	D73	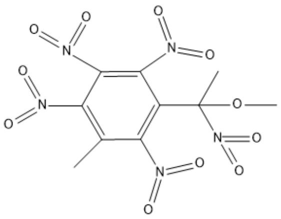

D30	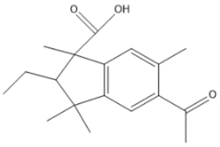	D74	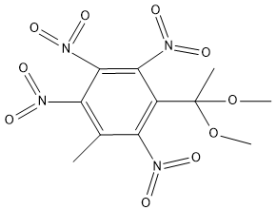
D31	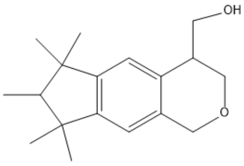	D75	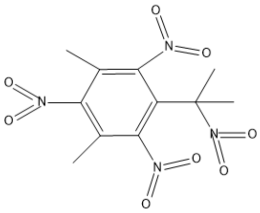
D32	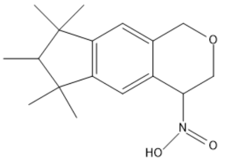	D76	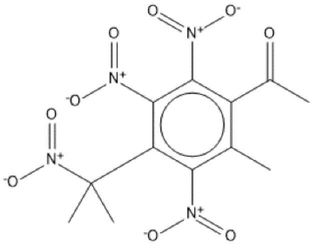
D33	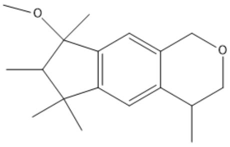	D77	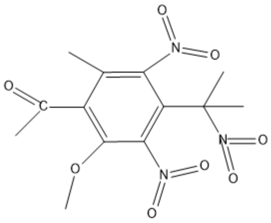
D34	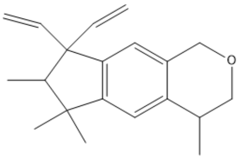	Phantolide	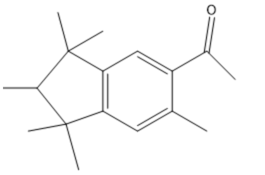
D35	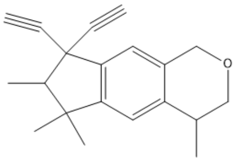	Celestolide	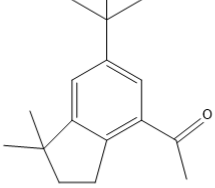
D36	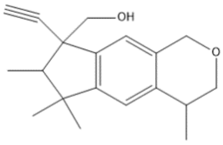	Tonalid	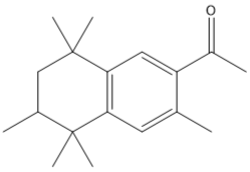

D37	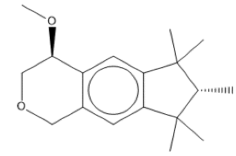	Galaxolide	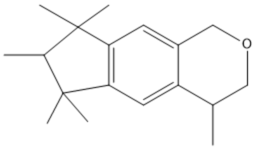
D38	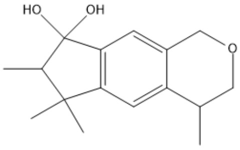	Versalide	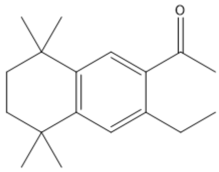
D39	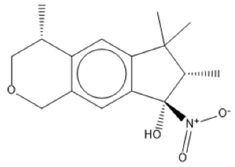	Musk xylene	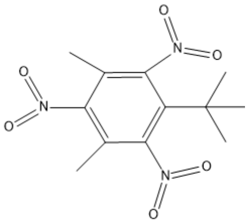
D40	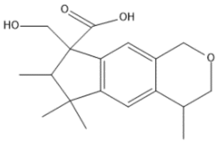	Muscone	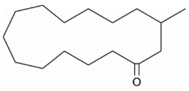
D41	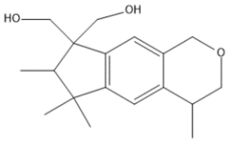	Musk methy	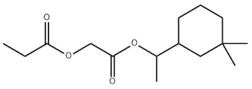
D42	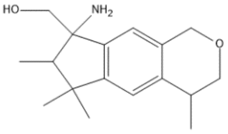	Musk ambrette	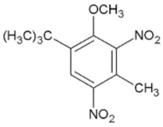
D43	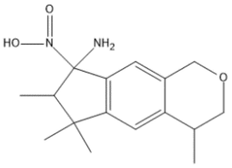	Moskene	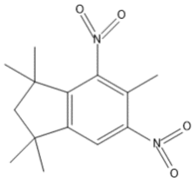
D44	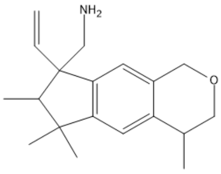	Musk ketone	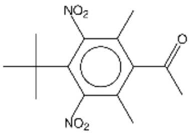

```python
import pandas as pd
import numpy as np
from scipy.stats import pearsonr
```

```python
dataSet = pd.read_excel("pi1.xlsx")
pearson_result=dataSet.corr()
pearson_result.to_excel("person.xlsx")
```

```python
def excuteFilter(x,pearson_result,dataSet,path):

    header=pearson_result.columns

    data = pearson_result.values

    indices = np.triu_indices_from(data)

    [rows, cols] = data.shape
    addlist = []
    removelist = []
    removeindex = []

    for i in range(rows):
        if header[i] not in removelist:
            for j in range(cols):
                if j < i:
                    if data[i,j]>x or data[i,j]<-x:
                        if header[j] in addlist:
                            if header[i] not in removelist:
                                removelist.append(header[i])
                                continue
                        if header[i] not in addlist:
                            addlist.append(header[i])
                        if header[j] not in removelist:
                             removelist.append(header[j])

    retainList = []
    for col in header:
        if col not in removelist:
            retainList.append(col)
            print(col)

    dataSet[retainList].to_excel(path,index = False)
```

```python
excuteFilter(0.6,pearson_result,dataSet,"retainDataSet.xlsx")
```

```python
import seaborn as sns
import matplotlib.pyplot as plt
```

```python
dataframe = pd.read_excel("retainDataSet.xlsx")
dcorr=dataframe.corr()
```

```python
from matplotlib import rcParams

plt.rcParams['font.family']=['Times New Roman']
plt.rcParams['axes.unicode_minus']=False
plt.mathtext(fontfamily= 'Times New Roman')
sns.set(font_scale=1.3)
plt.subplots(figsize=(20, 20))

fig=sns.heatmap(
    dcorr,
    cmap='YlGnBu',
    annot=True,
    fmt=".2f",
    mask=np.triu(np.ones_like(dcorr,dtype=bool))
)

fig_path="retainHeatmap.jpg";
heatmap = fig.get_figure()
heatmap.savefig(fig_path, dpi = 600)
```

Figure. S1 Processing code for Pearson correlation coefficient

```
In [1]: import numpy as np
        import pandas as pd
        from pulearn import BaggingPuClassifier
        from sklearn.tree import DecisionTreeClassifier
        from sklearn.ensemble import RandomForestClassifier
        from sklearn.svm import SVC
        pd.set_option('max_columns',1000)
        pd.set_option('max_row',300)
        pd.set_option('display.float_format', lambda x:' %.5f' % x)
        pd.set_option('display.max_rows',None)
        pd.set_option('display.max_columns',None)
        pd.set_option('max_colwidth',10000)
        import sys
        np.set_printoptions(threshold=sys.maxsize)

In [2]: df = pd.read_excel("PU1.xlsx",header = None)

In [3]: X=df.iloc[:,:-1]
        y=df.iloc[:,-1]

In [4]: for i in range(1,100):
            bc = BaggingPuClassifier(
                RandomForestClassifier(
                    n_estimators =5,
                    random_state =0,
                    criterion='gini',
                    min_samples_split=2,
                    max_features='auto',
                ),
                n_estimators =i,
                max_samples=sum(y),
                n_jobs=-1,
                random_state =0
        )
            bc.fit(X,y)
            score=bc.oob_score_
            print('i='+str(i)+'score',score)

...

In [9]: bc = BaggingPuClassifier(
              RandomForestClassifier(
                    n_estimators =5,
                    random_state =0,
                    criterion='gini',
                    min_samples_split=2,
                    max_features='auto',
                ),
            n_estimators =30,
            max_samples = sum(y),
            n_jobs = -1,
            random_state =0
        )
        bc.fit(X, y)

Out[9]: BaggingPuClassifier(base_estimator=RandomForestClassifier(n_estimators=5,
                                                                  random_state=0),
                            max_samples=11, n_estimators=30, n_jobs=-1, random_state=0)

In [10]: score=bc.oob_score_
         print(score)

         0.7045454545454546

In [11]: print(bc.oob_decision_function_)

...

In [12]: bc.predict_proba(X)

...
```

Figure. S2 Processing code for bagging-Random Forests

```
import numpy as np
import pandas as pd
from pulearn import BaggingPuClassifier
from pulearn import ElkanotoPuClassifier
from sklearn.tree import ExtraTreeClassifier
from pulearn import WeightedElkanotoPuClassifier
from sklearn.tree import DecisionTreeClassifier
from sklearn.ensemble import RandomForestClassifier
pd.set_option('max_columns',1000)
pd.set_option('max_row',300)
pd.set_option('display.float_format', lambda x:' %.5f' % x)
pd.set_option('display.max_rows',None)
pd.set_option('display.max_columns',None)
pd.set_option('max_colwidth',10000)
import sys
np.set_printoptions(threshold=sys.maxsize)
```

```
df = pd.read_excel("PU1.xlsx",header = None)
X=df.iloc[:,:-1]
y=df.iloc[:,-1]
```

```
for i in range(100,500):
    bc = BaggingPuClassifier(
        ExtraTreeClassifier(),
        n_estimators =i,
        max_samples=sum(y),
        n_jobs=-1,
        random_state =0,
        #verbose=i
)
    bc.fit(X,y)
    score=bc.oob_score_
    print('i='+str(i)+'score',score)
```

```
bc =BaggingPuClassifier(
    ExtraTreeClassifier(),
    n_estimators =416,
    max_samples = sum(y),
    n_jobs = -1,
    random_state = 0,
)
bc.fit(X, y)
```

```
BaggingPuClassifier(base_estimator=ExtraTreeClassifier(), max_samples=11,
                    n_estimators=416, n_jobs=-1, random_state=0)
```

```
print("model score:",bc.oob_score_)
```

```
model score: 0.7272727272727273
```

```
print(bc.oob_decision_function_)
```

...

```
bc.predict_proba(X)
```

Figure. S3 Processing code for bagging-Extremely Randomized Tree

```python
import numpy as np
import pandas as pd
from pulearn import BaggingPuClassifier
from sklearn.tree import DecisionTreeClassifier
from sklearn.ensemble import RandomForestClassifier
from sklearn.neighbors import KNeighborsClassifier
from sklearn.svm import SVC
from pulearn import ElkanotoPuClassifier
pd.set_option('max_columns',1000)
pd.set_option('display.float_format', lambda x:' %.5f' % x)
pd.set_option('display.max_rows',None)
pd.set_option('display.max_columns',None)
pd.set_option('max_colwidth',10000)
import sys
np.set_printoptions(threshold=sys.maxsize)
from sklearn.neural_network import MLPClassifier
from sklearn.ensemble import AdaBoostClassifier
from sklearn.ensemble import GradientBoostingClassifier
```

```python
df = pd.read_excel("PU1.xlsx",header = None)
```

```python
X=df.iloc[:,:-1]
y=df.iloc[:,-1]
```

```python
for i in range(50,500,50):
    bc = BaggingPuClassifier(
        GradientBoostingClassifier(
        n_estimators = 441,
        random_state=0,
        learning_rate=0.5
        ),
        n_estimators =i,
        max_samples=sum(y),
        n_jobs=-1,
        random_state =0
)
    bc.fit(X,y)
    score=bc.oob_score_
    print('i='+str(i)+'score',score)
```

```python
bc = BaggingPuClassifier(
    GradientBoostingClassifier(
        n_estimators = 441,
        random_state=0,
        learning_rate=0.5
    ),
    n_estimators =900,
    max_samples = sum(y),
    n_jobs = -1,
    random_state =0
)
bc.fit(X, y)
```

```
BaggingPuClassifier(base_estimator=GradientBoostingClassifier(learning_rate=0.5,
                                                              n_estimators=441,
                                                              random_state=0),
                    max_samples=11, n_estimators=900, n_jobs=-1,
                    random_state=0)
```

```python
score=bc.oob_score_
print(score)
```

```
0.7727272727272727
```

```python
print(bc.oob_decision_function_)
```

...

```python
bc.predict_proba(X)
```

...

Figure. S4 Processing code for bagging-Gradient Boosting
